# P1′ Residue-Oriented Virtual Screening for Potent and Selective Phosphinic (Dehydro) Dipeptide Inhibitors of Metallo-Aminopeptidases

**DOI:** 10.3390/biom10040659

**Published:** 2020-04-24

**Authors:** Michał Talma, Artur Mucha

**Affiliations:** Department of Bioorganic Chemistry, Wrocław University of Science and Technology, Wybrzeże Wyspiańskiego 27, 50-370 Wrocław, Poland; michal.talma@pwr.edu.pl

**Keywords:** organophosphorus compounds, peptide analogs, enzyme inhibitors, ligand–enzyme interactions, molecular modeling and docking

## Abstract

Designing side chain substituents complementary to enzyme binding pockets is of great importance in the construction of potent and selective phosphinic dipeptide inhibitors of metallo-aminopeptidases. Proper structure selection makes inhibitor construction more economic, as the development process typically consists of multiple iterative preparation/bioassay steps. On the basis of these principles, using noncomplex computation and modeling methodologies, we comprehensively screened 900 commercial precursors of the P1′ residues of phosphinic dipeptide and dehydrodipeptide analogs to identify the most promising ligands of 52 metallo-dependent aminopeptidases with known crystal structures. The results revealed several nonproteinogenic residues with an improved energy of binding compared with the best known inhibitors. The data are discussed taking into account the selectivity and stereochemical implications of the enzymes. Using this approach, we were able to identify nontrivial structural elements substituting the recognized phosphinic peptidomimetic scaffold of metallo-aminopeptidase inhibitors.

## 1. Introduction

Phosphinic dipeptides are generally acknowledged as transition-state analog inhibitors of metallo-aminopeptidases [1,2,3], a large and homogenous class of exopeptidases that specifically cleave the N-terminal amino acid residues of polypeptides and proteins and contain metal ion(s) in their active sites. Being ubiquitously distributed in subcellular organelles, cytoplasm, and cellular membranes throughout all of the biological kingdoms, these enzymes perform critical roles in physiological processes and diseases. In humans, they are responsible for processing peptides and proteins involved in, for example, angiogenesis, antigen presentation, cancer and metastasis, blood pressure regulation, and bacterial and protozoal infections [3,4,5]. The inhibitory potential of phosphinic dipeptides originates from two fundamental structural features that determine the competitive mechanism of binding, and govern the potency and specificity of the ligands. First, the presence of the central phosphinic functional group ensures steric and electronic resemblance to the scissile amide bond of hydrolyzed substrates in the tetrahedral transition state. This also involves the ability to coordinate with catalytic metal ion(s). Second, the optimized structures of the P1 and P1′ side chains guarantee complementarity to the corresponding S1 and S1′ binding pockets (encryptions according to the nomenclature proposed by Schechter and Berger to define the N-terminal, non-primed and C-terminal, primed residues of the scissile amide bond in peptide substrates, and the corresponding binding pockets [6]).

Mammalian, protozoan, and bacterial enzymes of the M01 and M17 families have probably been the most comprehensively studied metallo-aminopeptidases with phosphinic dipeptides [2,5]. Alanine aminopeptidase (aminopeptidase N, APN, M01.001, EC 3.4.11.2) and leucine aminopeptidase (LAP, M17.001, EC 3.4.11.1) can be considered as the prototypical representatives. Importantly, the greatest results of inhibition were achieved for compounds comprising side chains originating from nonproteinogenic amino acids. Such fragments of optimal ligands are not trivial and can hardly be predicted by directly translating the results of studies on peptidyl substrates. These residues can be conveniently designed with the aid of molecular modeling methods; however, this process may demand a few iterations of design, synthesis, and evaluation. For example, it took us ten years to inhibit (*K*_i_) mammalian APN and LAP by phosphinic dipeptides at a low nanomolar/subnanomolar level [7,8].

**Scheme 1:**
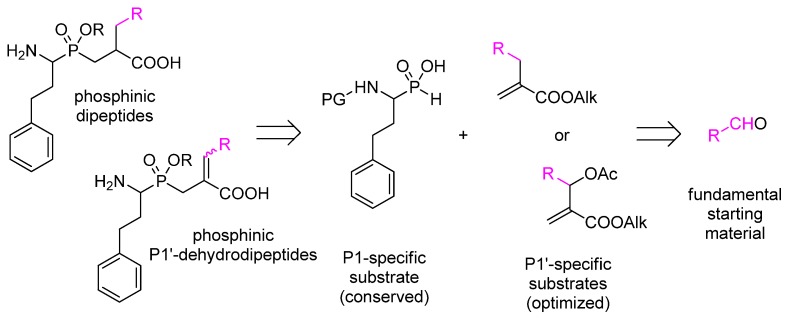
Retrosynthetic analysis of phosphinic dipeptide and dehydrodipeptide analogs. The P1 phenylethyl residue (homophenylalanine) is conserved as canonical. The pathways involve P1′-specific electrophiles that can be obtained from aldehydes.

The synthesis of each individually modified metallo-aminopeptidase inhibitor requires the development of multistep synthetic pathways with orthogonal protective strategies, leading to fundamental building blocks. In the most typical procedure, P1-specific N-protected aminoalkyl-H-phosphinic acid and P1′-specific α-substituted acrylate are such components (Scheme 1) [1]. Aldehydes, whose structures encode the P1 and P1′ residues, are primary substrates for the preparation of these synthons. Thus, the proper and reliable selection of the fundamental starting materials is of invaluable significance as it results in either fruitful or vain efforts.

In the current work, we address the design issue to a virtual screening approach. In particular, the P1′ residues of active and specific phosphinic dipeptide inhibitors of metallo-aminopeptidases were targeted. A total of 900 commercially available aldehydes were selected as potential precursors of these P1′ residues, while the P1 fragment (mimicking homophenylalanine) remained conserved. Docking analysis was performed with 52 crystal structures of 27 enzymes. In addition, P1′-dehydrodipeptide analogs were included in the study, as the aldehydes may also be the substrates of the P1′-unsaturated counterparts. One of the methods for their preparation involves substitution of P1-specific N-protected aminoalkyl-H-phosphinic acids with Morita–Baylis–Hillman acetates (Scheme 1) [9]. The latter are obtained by acetylation of allyl alcohols, which, in turn, are synthesized from aldehydes and alkyl acrylates. Similar to the saturated compounds, phosphinic dehydrodipeptides were reported as inhibitors of metallo-aminopeptidases, although to a lesser extent [10].

As the main outcome of this study, we defined a set of novel nonproteinogenic P1′ substituents that are potential structural fragments of active pseudodipeptide inhibitors of metallo-aminopeptidases. The details of interactions with the target proteins were analyzed at the molecular level by docking of the individual stereoisomers in the enzyme active sites. Stereochemical considerations allowed implementation of favorable configurations of the α-carbon atoms and geometry of the double bonds in the optimized structures.

## 2. Materials and Methods

### 2.1. Target Selection and Preparation

The metallo-aminopeptidases from the MEROPS database were selected for this study (ebi.ac.uk/merops/) [11]. The fundamental criterion was an available crystal structure in the Research Collaboratory for Structural Bioinformatics Protein Data Bank (RCSB-PDB, rcsb.org) [12]. For the enzymes with more than one deposited structure for a particular species, the structure with the highest resolutions was chosen. Ten of 62 crystal structures downloaded from the PDB lacking either metal ion(s) in the active centers or putative peptidases were excluded from the study. The remaining 52 enzymes are listed in Table 1. Using options implemented in Protein Preparation Wizard the structures were prepared for calculation: all of the water molecules and the ligands were removed, and the proteins were protonated at the experimental pH [13].

### 2.2. Ligand Preparation

**Figure 1:**
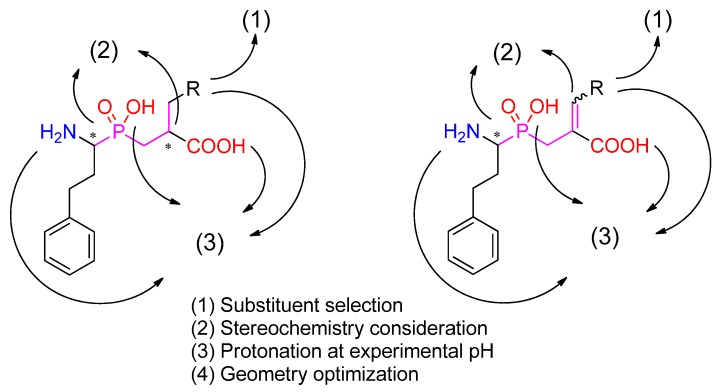
A general procedure for phosphinic ligand preparation. Following the R substituent selection, four possible stereoisomers (either *R/S, R/S* or *R/S, E/Z* combinations) were considered in the calculations.

The first step of structural optimization of phosphinic dipeptide and dehydrodipeptide inhibitors (Figure 1) involved selection of aldehydes as the building blocks coding the R. The set of commercially available carbonyl compounds was taken from the Sigma-Aldrich/Merck online catalog. The full list of the aldehydes in this study is included in the Supplementary Materials. LigPrep [66] was used to create the stereoisomers of the final compounds and to pronate them at the experimental pH. Epik and OPLS3e force fields were used for the geometry optimization, and the metal binding states were added to the calculations [13]. The procedure was also applied to known inhibitors, which were considered as the reference compounds.

### 2.3. Docking Studies

**Figure 2:**
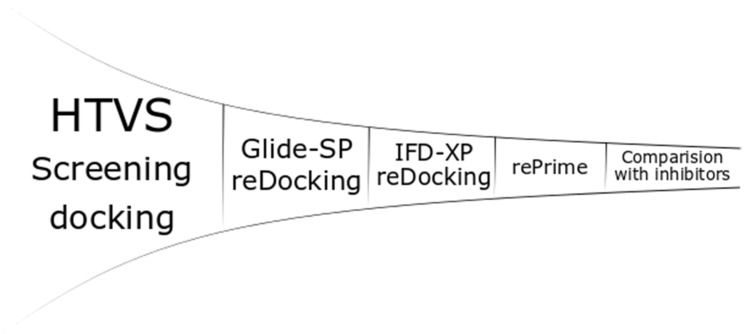
Generalized docking scheme. HVTS, high throughput virtual screening; SP, standard precision; IFD, induced fit docking; XP, extra precision.

All of the stereomeric compounds were docked (Figure 2) in the grid created for the selected enzymes (900 × 4 × 52 experiments). Receptor Grid Generation used the centroid position of metal or metals for site creation. The site of the enclosing box was set at 20 Ȧ (36 Ȧ for PDB: 2EK8), and all of the rotatable groups of amino acids in the range were selected. Other settings were set to default, and no constraints or excluded volumes were added. The ligands were docked by Glide-HTVS (High Throughput Virtual Screening algorithm) with flexible ligands, nitrogen inversions and ring conformation sampling. Bias sampling of torsions was performed for the amides only. The Epik state penalties were added to the docking score. Five ligand poses were included in scoring optimization, but the pose with the highest score was written out.

The top 100 inhibitors with the highest Glide-HTVS docking score were selected for the next step: redocking with the Glide-SP (standard precision) algorithm. The settings were kept the same as in the previous step. The top 10 inhibitors interacting with metal ion(s) by the phosphinic group were conservatively used in the next step. The inhibitors that did not interact with metal(s), but received relatively high scores were also included (maximum of 10). Thus, the number of inhibitors ranged from 10 (only phosphinic) to 20 (all top 10 metal-interacting inhibitors with relatively low scores compared with the top 10 nonmetal-interacting inhibitors). The penultimate stage was induced fit docking (IFD) [67]. The box center was set on the center of the metal or metals with size of 20 Ȧ to keep the size similar to the grid from the first step (36 Ȧ for PDB: 2EK8). The VSGB (variable-dielectric generalized born) model, which incorporates residue-dependent effects, was used. The solvent was water. The side chains were optimized within 5.0 Ȧ of ligand poses, and Glide redocking was carried out with the XP (extra precision) algorithm. The top pose for each ligand was saved. The last steps, rePrime refinement [67] and MM-GBSA (molecular mechanics-generalized born surface area) calculations, were performed to calculate the Gibbs free energies with protein flexibility, and the distance from the ligand was also set to 5.0 Ȧ.

### 2.4. ADMET Studies

ADMET was performed online with SwissADME [68] online tools. The following properties were selected for publication: molecular weight (MW), lipophilicity score (logP), water solubility (WS), number of possible H-bond donors (H_don_) and acceptors (H_acc_), Lipinski’s properties for drug likeness, and gastrointestinal absorption (GI).

## 3. Results and Discussion

As the main outcome of the screening, the top results in three categories are presented and discussed. The optimized structures emerged based on the lowest calculated Gibbs free energy of ligand–protein complexes, taking into account compound stereochemistry. The first category covered potentially the most active phosphinic (dehydro)dipeptide inhibitors. In the cases of enzymes for which the most favorable ligand did not interact with the metal ion(s), the best metal-complexing inhibitors were also selected. The structures that were specifically bound were also proposed as the third category. The specificity reflected with the particular P1′ substituent presents exclusively among the top 10 inhibitors of a single aminopeptidase.

The binding energies obtained for virtual structures were compared with the values calculated for potent known inhibitors that were reported in the literature, and the most frequent inhibitor was bestatin. Only selected data are included in the body of the main manuscript. The M1 family, the most numerous and intensively studied family, is presented in detail. Then, the results for arbitrarily chosen enzymes important for medicinal chemistry applications are also given. The full list of preferred inhibitors for each enzyme is given in the Supplementary Materials (Table S1).

### 3.1. M1 Family

M1 is a family of metallo-aminopeptidases containing the ‘HEXXH-(X_18_)-E’ zinc-binding motif and ‘GXMEN’ exopeptidase motif in their sequence and one zinc cation in their active site [3,69]. The family contains enzymes widely occurring in living organisms, including alanyl, cystinyl, and lysyl aminopeptidases; leukotriene A4 hydrolase; and endoplasmic reticulum aminopeptidases I and II [70]. These enzymes play different physiological roles and are also targets for treating various pathological states. Nine M1 aminopeptidases have been identified in humans, six of which have been associated with disorders [69]. For example, their altered activity has been connected with regulation of blood pressure controlling systems, resulting in hypertension and strokes [71]. M1 aminopeptidases are also involved in cancer development [69]. In particular, APN has been shown to be overexpressed in leukemia blasts in acute myeloid leukemia. APN is active in tumor angiogenesis and metastasis [72]. LTA4H has been associated with acute lung damage, while dysregulation of ERAP1, ERAP2, and IRAP can reflect the risk of autoimmune diseases. M1 aminopeptidases are receptors for viruses, for example, coronaviruses [73]. They are also connected with several infections caused by bacteria, for example, meningitis caused by *Neisseria meningitides* [74], or protozoans, for example, malaria transmitted by *Plasmodium falciparum*. The parasite aminopeptidase of this species is involved in the digestion of short hemoglobin-derived peptides and has been rated as a highly prospective therapeutic target [75,76].

The S1′ binding pockets of M1 aminopeptidases are available for substrates with a wide range of sizes (Table 2). Principally, these cavities are formed from functional (performing catalytic roles) and hydrophobic amino acid residues. Among the functional amino acids, each enzyme comprises tyrosine, which stabilizes the *gem*-diol transition state, and histidine, which participates in metal ion complexation. The carbonyl oxygen atom of the P1′ residue of the cleaved substrates interacts with the positively charged side chains of arginine and lysine and noncharged chains of asparagine and threonine or the amino group of amino acids located on the border of the S1–S1′ cavities. Acidic side chains of glutamic and aspartic acids are responsible for controlling the flexibility of the active site. The position of these amino acids may change inside or outside the cavity.

In accordance with the overall character of S1′ pockets, the best docking scores for M1 aminopeptidases were achieved for bulky aromatic and heteroaromatic P1′ substituents (Table 3). Although π-conjugated systems are naturally hydrophobic, certain modifications with heteroatoms/functional groups tighten binding with the enzyme active site by making specific contacts. In some cases of sterically extended residues, the fit appeared so close that the S1′ pocket seemed virtually “stoppered” in docking experiments. Rotational freedom in phosphinic dipeptides positively influenced the energy of binding. These analogs were more frequently represented among the top compounds compared with their P1′-unsaturated counterparts. Nevertheless, a couple of enzymes appeared to favor dehydropeptides among the dozen listed in Table 3. Regarding stereochemistry, the *R*,*S* diastereoisomers of phosphinic dipeptides prevailed, and this result is consistent with the relative configuration l,l of natural substrates. For the dehydro analogs, the *E* geometry was somewhat favored.

**Figure 3:**
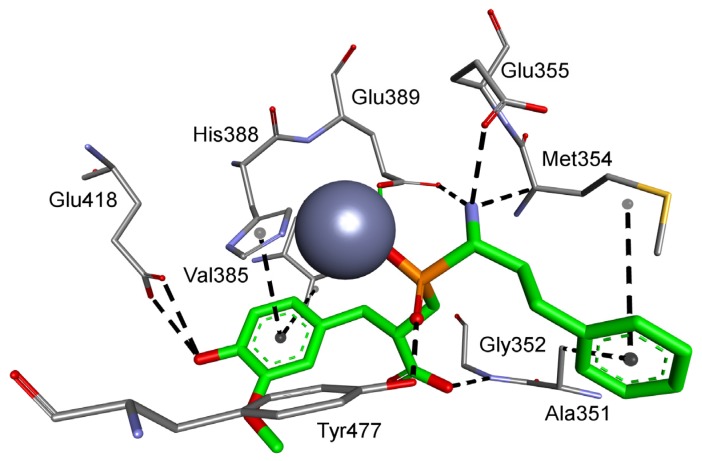
Modeled binding mode of the phosphinic dipeptide analog containing a 4-hydroxy-3-methoxyphenyl P1′ fragment in the active center of human aminopeptidase N (Protein Data Bank (PDB): 4FYT). Amino acid residues of the inhibitor and enzyme are shown as sticks, while zinc ions are shown as gray spheres. Hydrogen bonds and nonpolar interactions are shown as thin dashed lines.

Considering the selected examples, human APN showed the minimal energy of binding with phosphinic dipeptide derived from vanillin (R = 4-hydroxy-3-methoxyphenyl) (Figure 3), yet the compound could not be considered specific. Phosphinic inhibitors with P1′ aromatic rings functionalized with hydroxyl groups have been recognized as good inhibitors of metallo-aminopeptidases [7], as OH in the *para* position is well positioned to form hydrogen contacts with the carboxylate of Glu418. Flexibility of the side chain directs the aromatic ring towards Val385 and His388. Stacking of the ring with imidazole is facilitated by oxygen-containing groups, which change its character by the electron-withdrawal effect. The distance of 4.04 Å to the aromatic ring of Tyr477 causes additional stabilization through hydrophobic interactions. The C-terminal carboxyl group is pointed to Gly352. The compound has a nonnatural configuration on the α carbon of the P1 fragment. This is not significant, as both arrangements gave rise to very similar inhibition potential. It is also typical to observe the 2-phenylethyl fragment of hPhe-based inhibitors; because of its high flexibility, the absolute configuration does not determine the activity [84]. The most specific compound, whose structure is based on the 5-amino-thiophen-2-yl residue, reproduces an interaction with Glu418 (involving NH_2_), but the heteroaromatic ring is less conveniently positioned compared with His388 (Figure S1). The P1′ fragment is brought closer to the surface of the cavity, strengthening the influence of Thr384 and Val385. To our satisfaction, the binding energy calculated for a phosphinic dipeptide that was rationally designed, synthesized, and described in our previous studies (*K*_i_ = 0.69 nM) outscored the current structures [8]. However, starting materials with the specific P1 fragment (4-aminomethylphenyl) are not commercially available, and compound development demands a complex and multistep preparation. This can be considered an exception. In general, the structure proposed here surpasses known inhibitors.

As the overall architecture of the S1′ binding sites of porcine, bacterial, and protozoal APNs is rather conserved, the advantageous P1′ residues of these peptidases appeared similar to those of the human counterpart. They all contained two oxygen atoms at the remote positions of the phenyl ring. The oxygen atoms come from either boronic acid functionality (porcine APN) or 3,4-hydroxy/methoxy groups (*E. coli* and *P. falciparum* APNs). Interestingly, the bacterial enzymes showed a preference towards the constrained structure of phosphinic dehydrodipeptides. The energy of binding for this compound was equal to a very potent known pseudotripeptidyl inhibitor [79].

**Figure 4:**
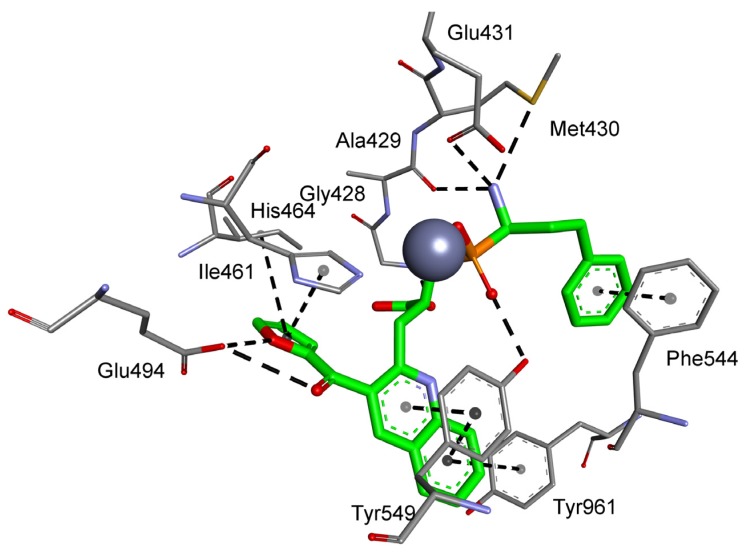
Modeled binding mode of a phosphinic dipeptide analog containing a 3-(furan-2-carbonyl)quinolin-2-yl P1′ fragment in the active center of cystinyl aminopeptidase (PDB: 5MJ6).

Groups that may be used in the development of molecular probes, including 3-(furan-2-carbonyl)quinolin-2-yl, 6-ethyl-4-oxo-4*H*-chromen-3-yl, phenanthren-4-yl, and quinolin-4-yl, are well scored for particular enzymes. Thus, they have the potential to serve as substituents with two roles as inhibitors and affinity-based probes. One of the most exciting results from the binding energy calculations was achieved for a 3-(furan-2-carbonyl)quinolin-2-yl-based inhibitor of cystinyl aminopeptidase. The obtained energy value was double that calculated for a very potent ligand. The residue tightly fits to the S1′ pocket and fully fills the cavity (Figure 4). The quinoline ring is situated between two aromatic rings of Tyr549 and S1 Tyr961, which highly stabilize the structure. The furan ring is located near His464, Ile461, and Glu494 at the opposed wall of the pocket.

The quinoline system installed on the dehydropeptide scaffold was shown to be well accepted by aminopeptidase from *N. meningitidis*. The ring is centrally located between aromatic residues of Tyr377 and His293, and these interactions cover the opposing walls of the S1′ pocket (Figure S2). The specific biphen-2-yl inhibitor of a comparable size is shifted more to the bottom to form numerous hydrophobic contacts with Phe271, Tyr372, and Tyr377 and long-range contacts with Val290 (Figure S3).

**Figure 5:**
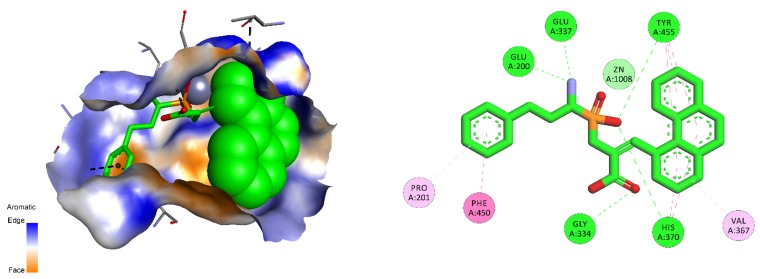
Modeled binding mode of a phosphinic dehydrodipeptide analog containing a phenanthren-4-yl P1′ fragment in the active center of ERAP2 aminopeptidase (PDB: 5AB0), with specified interactions as dashed lines: hydrogen bonds (green), π-alkyl (light pink), π–π stacking (dark pink).

The aminopeptidase ERAP2 also prefers spatial aromatic P1′ substituents; however, these bulky residues alter the canonical binding mode. The lowest energy inhibitor is supposed to interact with the metal ion by the C-terminal carboxyl group, not by the phosphinic moiety (Figure S4). The two aromatic rings of 2-benzyloxyphenyl surround Tyr455 owing to the flexibility of the methoxy connection. This limits the opportunity for typical complexation. Similarly, the most specific phosphinic dipeptide bearing a diphenylmethyl residue does not employ the central phosphinate to coordinate with the zinc atom (Figure S5). Strong stacking interactions between aromatic systems shift the overall ligand–protein architecture. One of the P1′ phenyl rings is inserted between tyrosines 455 and 892 to form a sandwich structure, while the other one occupies the bottom-central position of the S1′ pocket between Tyr892 and Trp363. Such a bulky group as phenanthrene can also be accepted in the P1′ position. This aromatic system substituting the dehydrodipeptide scaffold of the *Z* configuration forms a constrained system that perfectly fills the corresponding cavity (Figure 5). Despite a visibly higher energy compared with the abovementioned cases, these compounds preserve phosphinate–zinc complexation abilities.

Large S1′ cavities could also be effectively explored by alkylaryl substituents. *tert*-Butyl groups were found to be particularly advantageous for aminopeptidase from *S. cerevisiae* (3,5-di-*tert*-butylphenyl) and cold-active aminopeptidase (2-*tert*-butylthiophenyl). In the second case, the sulfur atom participates in the interaction with Lys586, while the alkyl wraps around Tyr410 (Figure S6). The structure of the phosphinic dipeptide based on this P1′ substituent can be promising because of the low energy calculated for the ligand–protein complex, which is accompanied by a high selectivity.

For particular aminopeptidases with relatively small S1′ binding pockets, namely aminopeptidase A and human leukotriene A4 hydrolase, ambiguous results were obtained. Aminopeptidase A prefers substrates with acidic amino acid residues, whereas the best computed structures comprise a positively charged nitrogen atom. The docking results revealed the possibility of nontypical binding. Phosphinic dipeptide with a 3-isobutylaziridin-2-yl P1′ fragment may interact with the zinc ion via a phosphinate oxygen atom, but both side chains are presumably located on the surface of the S1′ pocket (Figure S7). The most specific derivative (comprising 1-methyl-1*H*-imidazol) binds in a reverse direction. Although the phosphinate moiety maintains the central position, the P1′ residue occupies the S1 cavity, and vice versa. The calculated binding energies are not remarkable. For leukotriene A4 hydrolase, none of the docked inhibitors exhibit canonical metal complexation. In the case of the best inhibitors, this ability could be assigned to the boronyl group of the P1′ substituent.

In conclusion, for M1 metallo-aminopeptidases, energetically favored results were obtained for compounds with large side chains filling the active center. Interactions with the amino acids on the borders of consecutive pockets are also of great importance, in particular, for catalytic Tyr and His residues involved in ligand binding.

### 3.2. Human Aminopeptidases other than M1

Metallo-aminopeptidase activity in humans involves extensive catalytic machinery working with different types of peptides. It is indispensable to produce vast amounts of enzymes that can cope with this task under different conditions. The M1 metallopeptidase family is 1 of 29 formally classified families and one of only three families in which the crystal structures of human enzymes were determined (Table 1). The other two families are M18 and M24. The functions of human M18 aspartyl aminopeptidase are still poorly understood. It participates in blood pressure regulation and maintenance of proper hydroelectrolytic balance, and it has also been associated with angiogenesis in breast cancer [85]. The active center of M18 aspartyl aminopeptidase contains two metal ions and is open to the central tunnel formed of 12 subunits. This gives the protein the opportunity to bind long peptides in a number of consecutive pockets. The S1 pocket is arranged to bind asparagine or glutamine, while the S1′ pocket prefers small alkyl chain-type amino acid residues (Table 4). Human M24 aminopeptidases can be divided into two subgroups, namely methionine metallo-aminopeptidases and Xaa-Pro aminopeptidases. The two subgroups are differentiated based on the presence or absence of a common fold called the “pitta bread”. Similar to other aminopeptidases, their substrate specificity is broad [86]. The catalytic centers contain two cobalt or manganese ions. The enzymes differ in amino acid preferences and active site architecture (Table 4). For example, Xaa-Pro aminopeptidase 1 has a very small pocket specific for proline, while Xaa-Pro aminopeptidase 3, despite playing a similar function, has evolved to accept larger substrates.

The best phosphinic dipeptide inhibitors found for human M18 and M24 aminopeptidases appeared unexpectedly extended considering the limited size of the binding pockets (Table 5). The detailed molecular modeling analysis revealed that none of these compounds were able to bind in a substrate-like manner. For example, nominal P1′ substituents found for aspartyl aminopeptidase (3,4-dibenzyloxyphenyl), methionyl aminopeptidase 2 (4-borono-2,3-difluorophenyl), and aminopeptidase P3 (3-phenylaziridin-2-yl) had a tendency to occupy the S1 pockets, not the S1′ pockets, showing a distorted binding mode. This distortion involved interactions of the carboxylate group with the metal ions, and the C-terminal position was held by the phosphinate. The S1 cleft of aspartyl aminopeptidase was able to adopt unexpectedly bulky polyaromatic residues, which were surrounded by the alkyl residues of Lys358, Leu405, Val407, Pro412, Cys413, and His359 (π–π stacking) (Figure S8). Tyr381 formed a hydrogen bond with an ether oxygen atom. For aminopeptidase P3, 3-phenylaziridin interacted with the catalytic residues Pro301 and Asp331 (NH of aziridine) and His413 and Arg438 (Figure S9). For methionyl aminopeptidase 2, the ligand did not typically point towards Met384, but docked in the shallow part of the S1 cleft, close to catalytic amino acids (Figure S10). The phenyl ring and fluorine atoms interacted with His231 and His382, while the boronyl group formed numerous hydrogen bonds with Ser224, Lys249, and Gln457. In all three discussed cases, the P1 phenylethyl fragment was nonspecifically arranged.

**Figure 6:**
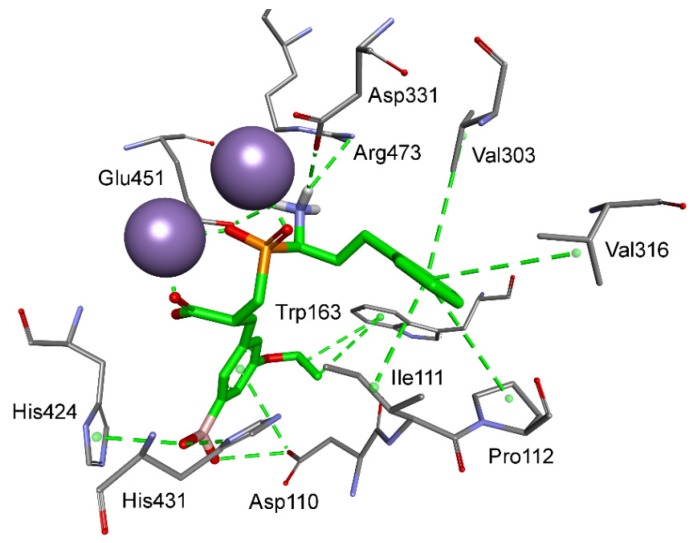
Modeled binding mode of a phosphinic dipeptide analog containing a 3-borono-5-isopropoxyphenyl P1′ fragment in the active center of aminopeptidase P3 (PDB: 5 × 49). Amino acid residues of the inhibitor and enzyme are shown as sticks, while cobalt ions are shown as purple spheres. Hydrogen bonds and nonpolar interactions are shown as thin dashed lines.

Aromatic residues functionalized with the boronyl group appeared the most universal in the construction of the best inhibitors of M18 and M24 aminopeptidases in different categories. As mentioned, the 4-borono-2,3-difluorophenyl substituent of phosphinic dipeptide was the most energetically beneficial for methionyl aminopeptidase 2, whereas the 4-boronylphenyl group of the dedydrodipeptide scaffold was the most beneficial for methionyl aminopeptidase 1, and the 4-borono-2-fluorophenyl-modified phosphinic dipeptide was the most beneficial for aminopeptidase P1. Borono-functionalized fragments were also present among the most specific or the best metal-interacting compounds. However, boronic acid was the most frequently engaged in metal complexation (together with the carboxylate, or alone), which caused a non-classical inclusion of this portion out of the S1′ clefts. Only individual borono-inhibitors exhibited the assumed binding modes, for example, 3-borono-5-isopropoxyphenyl phosphinate with aminopeptidase P3 (Figure 6). The P1′ acidic system of the ligand formed hydrogen bonds with His424, His431, and Arg438. The isopropyl group interacted with Trp163, while the ether oxygen atom was in hydrogen bond distance to the His421 amino group.

**Figure 7:**
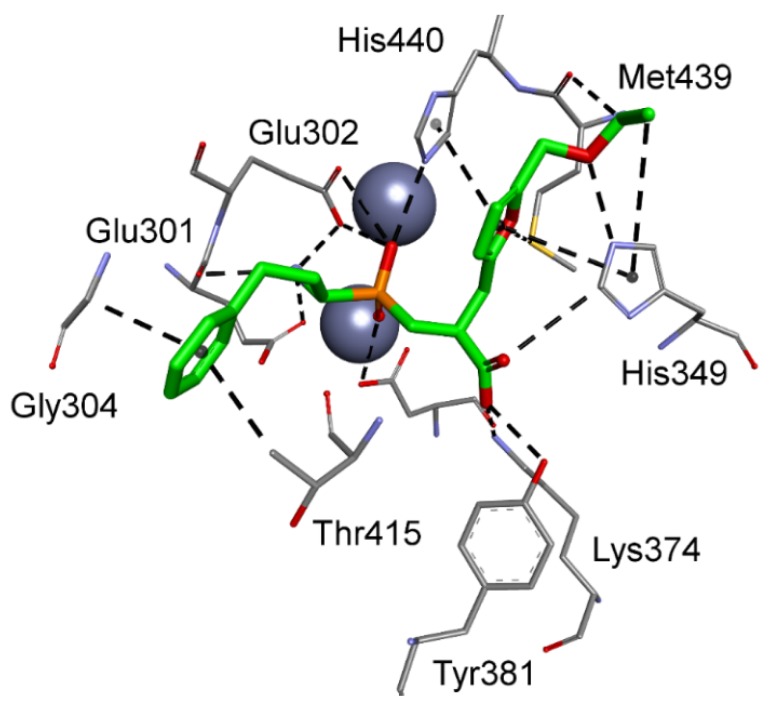
Modeled binding mode of a phosphinic dipeptide analog in the active center of aspartyl aminopeptidase (PDB: 4DYO) with the reverse orientation and a 5-(ethoxymethyl)furan-2-yl fragment (nominally P1′) extended to the S2 pocket.

Alterations were also observed for modes that involved metal–phosphinate coordination. 5-Ethoxymethylfuran-based phosphinic dipeptide inhibitor of aspartyl aminopeptidase and 4-(6-(hydroxymethyl)pyridinyl)phenyl-based methionyl aminopeptidase 1 showed, for instance, a reversed orientation of binding, with the P1 and P1′ positions flipped. For the first-mentioned case, the flexible N-terminal homophenylalanine fragment easily fit into the S1′ subsite, maintaining the ability of the amino group to interact with acidic residues of the zinc complexation system, in particular Glu301 and Glu302 (Figure 7). The phosphinic group coordinates with the metal cations, while the C-terminal carboxylate occupies the position of the acidic side chain of substrates preferred in the S1 pocket. The 5-ethoxymethylfuran group is extended to the S2 part and forms contacts with His349, Met439, and His440.

### 3.3. Aminopeptidases from Pathogenic Bacteria

Various pathological conditions can be caused by invading (micro)organisms, usually bacteria, protozoans, or viruses, that colonize humans and other species. These pathogens disrupt the body homeostasis through many mechanisms; from introducing toxins and competing for substrates to digesting peptides playing a significant role in the host organism. Microbial peptidases, including metallo-aminopeptidases, were adapted for the last-mentioned purpose [92]. They have been classified in a wide spectrum of families, but here, we focus on a few notorious examples in humans. Disabling their activity is extremely difficult because of the similarities in the active center structure and substrate specificity of microbial peptidases and human metallo-aminopeptidases. On the other hand, finding specific inhibitors offers specialized tools for highly targeted antimicrobial intervention.

The crystal structures of enzymes of microorganisms pathogenic to humans were determined in six families: M1, M17, M18, M24, M29, and M42. The M1 family (Table 3) includes, for example, the enzyme from *Plasmodium falciparum*, the most fatal of the four protozoan species that cause malaria in humans. This species also produces an M17 aminopeptidase [93], an aspartyl aminopeptidase classified in the M18 family [36], and the M24 aminopeptidase P [51]. Metallo-aminopeptidases are etiological factors of microbial infections, for example, meningitis caused by *Neisseria meningitides*, or yeast species. Accordingly, *Saccharomyces cerevisiae* can cause alterations in the production of B4 leukotriene and is a target in the treatment of bowel diseases, candidiasis, or psoriasis [94]. The full description of the active centers and substrate specificities of some microbial metallo-aminopeptidases is not always possible because of the lack of exhaustive data (Table 6).

**Figure 8:**
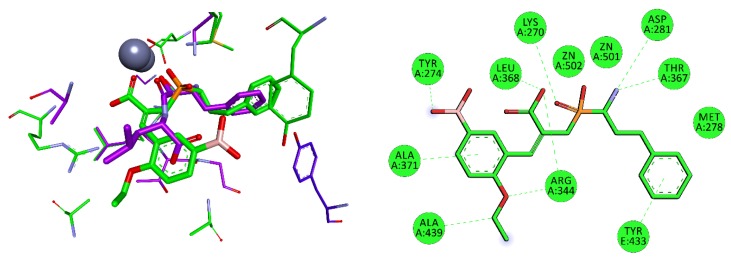
Modeled binding mode of a phosphinic dehydrodipeptide analog containing a 3-borono-6-ethoxyphenyl P1′ fragment in the active center of *Helicobacter pylori* aminopeptidase A/I (PDB: 4ZLA). The arrangement of the designed inhibitor was compared with that of bestatin, whose carbon atoms are colored in purple. Similar coloration was applied to amino acids interacting with the selected ligand. Carbon atoms of Tyr433 of another protein subunit are colored in dark blue. Amino acid residues of the inhibitor and enzyme are shown as sticks, while zinc ions are shown as gray spheres. Hydrogen bonds and nonpolar interactions are shown as thin dashed lines.

M17 mixed type A/I aminopeptidase from *Helicobacter pylori*, in addition to urease (Nobel Prize in Medicine for Marshall and Warren in 2005 [95]), is among the potential targets for organophosphorus inhibitors. This bizinc-dependent enzyme has a wide active center arranged for peptides with long side chains [51], but the S1′ preferences have not yet been specified. Nevertheless, good docking results were obtained for compounds with boronyl groups (Table 7). The 3-boronyl-6-ethoxyphenyl substituent proved the best dehydrodipeptide analog inhibitor, with a binding mode similar to that of bestatin (Figure 8). The P1 group of hPhe is arranged in S1 and interacts with Met278 and Tyr433 of another enzyme subunit. The P1′ substituent also fills the corresponding cavity very well. Tyr274 interacts with the boronyl group by a hydrogen bond, while the aromatic ring and ethoxy group form hydrophobic interactions. Lys270 plays a role in stabilizing the transition state of peptide bond hydrolysis; accordingly, it binds with its amino group to the central phosphinate moiety.

**Figure 9:**
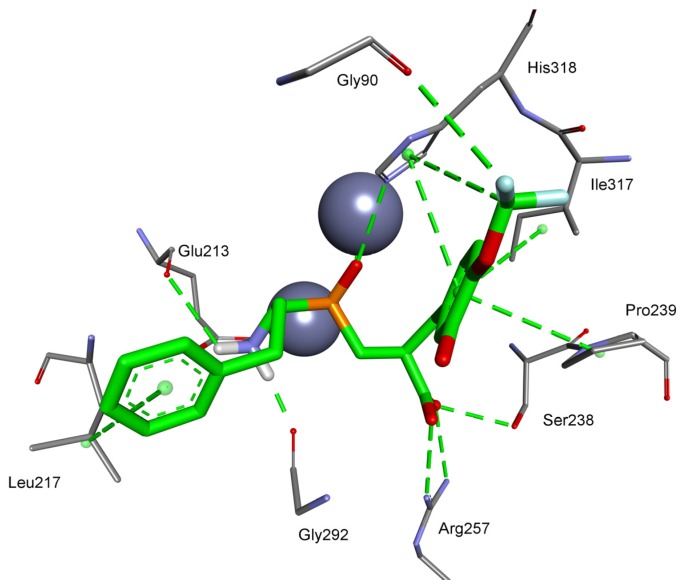
Modeled binding mode of a phosphinic dipeptide analog containing a 4-(difluoromethoxy)-3-hydroxyphenyl P1′ fragment in the active center of *Streptococcus pneumoniae* glutamyl aminopeptidase (PDB: 3KL9). Amino acid residues of the inhibitor and enzyme are shown as sticks, while zinc ions are shown as gray spheres. Hydrogen bonds and nonpolar interactions are shown as thin dashed lines.

The M17 enzymes are also characteristic of bacteria of the *Pseudomonas* genus, which are responsible for endogenous infections of individuals with reduced immunity, including pneumonia, among other microorganisms that express metallo-aminopeptidases and are involved in respiratory diseases, for example, *Pseudomonas aeruginosa* (M18), *Mycobacterium tuberculosis* (M24), or *Streptococcus pneumoniae* (M42). The best pseudodipeptide that interacts with metal ions of glutamyl M42 aminopeptidase via phosphinate functionality is based on the 4-(difluoromethoxy)-3-hydroxyphenyl P1′ fragment. Its orientation is reversed in the active center, similar to the aspartyl-specific enzyme (Figure 9). The C-terminal carboxylate is exposed to Arg257 and Ser238. The P1′ aromatic ring fits perfectly in the S1 pocket by multiple hydrophobic interactions with Pro239, Ile317, and catalytic His318. The docking is strengthened by additional halogen bonding of carbonyl Gly90.

**Figure 10:**
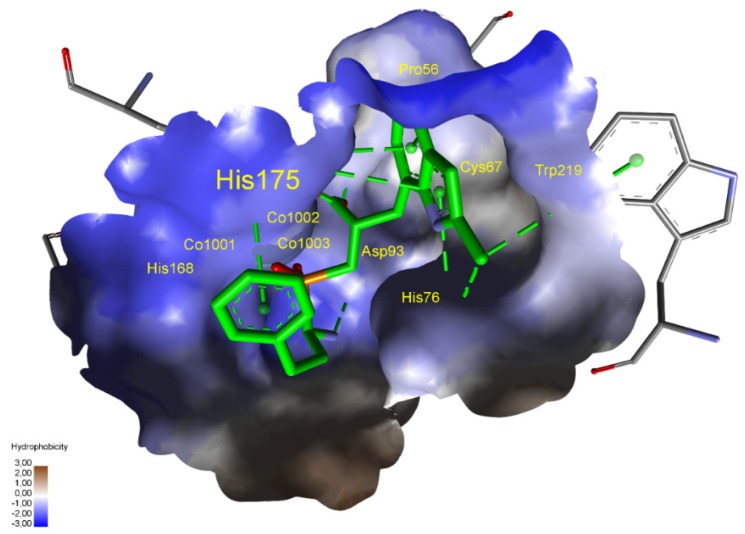
Modeled binding mode of a phosphinic dipeptide analog containing a 2-methylindol-7-yl P1′ fragment in the active center of *Streptococcus pneumoniae* glutamyl aminopeptidase (PDB: 3KL9). Amino acid residues of the inhibitor and enzyme are shown as sticks, while cobalt ions are shown as gray spheres. Hydrogen bonds and nonpolar interactions are shown as thin dashed lines. The surface is colored according to the hydrophobicity of amino acid side chains.

The situation for methionine aminopeptidase 1 from *Staphylococcus aureus* is complex as three cobalt ions in the active center are responsible for catalytic functions. This bacterium is responsible for purulent infections of the skin, subcutaneous and soft tissues, systemic infections, and poisoning associated with the production of specific toxins. The ΔG binding energies of predicted phosphinic dipeptide inhibitors are significantly lower than those of known ligands. For metal–phosphinate-interacting compounds, the large catalytic center allows both acidic groups to point towards the three cobalt regions (Figure 10). The spatial hydrophobic S1 pocket shows a preference towards the large, heteroaromatic system. Both aromatic rings of the ligand interact with abundant histidine residues.

## 4. Conclusions

The study demonstrated a noncomplex, practical, and inexpensive method of determining favorable P1′ substituents in the structure of phosphinic dipeptide inhibitors of metallo-aminopeptidases. The approach involved virtual screening of the inhibitors with commonly available molecular modeling tools. The optimized residues were not generated computationally, but were taken from the structures of commercially available synthetic precursors. This supposition should ensure the feasibility of the subsequent synthesis. The obtained dataset was analyzed considering stereochemical implications and selectivity of interactions in the enzyme active sites. The most potent, the most potent metal-interacting, and the most selective diastereomeric inhibitors that were calculated (by the lowest free binding energy) were thus identified. The structures of the optimized substituents appeared not evident and, quite surprisingly, not similar to those of proteinogenic residues. Two types of these structures were predominantly present. The first type included spatially extended P1′ fragments of aromatic character, principally hydrophobic, such as aryl, heteroaryl, and arylakil. These residues were characterized by extremely tight insertion into the large S1′ pockets. Substituents of a moderate size, but modified with two oxygen atom-containing group(s), for example, boronyl and dihydroxyl, represented the second most abundant group. Oxygen-comprising groups tended to form a favorable net of hydrogen bonds with the S1′ residues of certain metallo-aminopeptidases. However, the presence of alternatives to phosphinate metal binding groups, such as boronyl and C-terminal carboxyl groups, gave rise to alterations in the typical substrate-like binding mode. Such changes were the most frequent for aminopeptidases with small substrate-oriented S1′ subsites. Reorientation of the overall direction of binding was also observed in some cases. Enriched by such binding mode details, the method can be recommended as general to design specified residues of inhibitors of peptidomimetic structures.

Phosphinic dipeptides do not show toxicity [5] and meet the basic rules that would make them orally active drugs in humans. Selected ADMET properties predicted for the inhibitors of M1 aminopeptidases validate their druglikeness (Table 8, the full table is included in Supplementary Information, Table S2). Lipinski’s rule of five is fulfilled, as the compounds are relatively small (MW only in rare cases exceeds 500) and moderately lipophilic (logP from −1.6 to 3.3). The pseudopeptidic phosphinic backbone is a source of four hydrogen bond acceptors and one donor. These basic numbers may be increased for the heteroatoms comprised by the substituents. Water solubility and gastrointestinal absorption are variable. In summary, in addition to the biological activity, phosphinic dipeptide analogs possess the physicochemical properties needed for drug candidates, opening opportunities to use phosphinic dipeptide analogs in the treatment of a spectrum of diseases.

**Figure 11:**
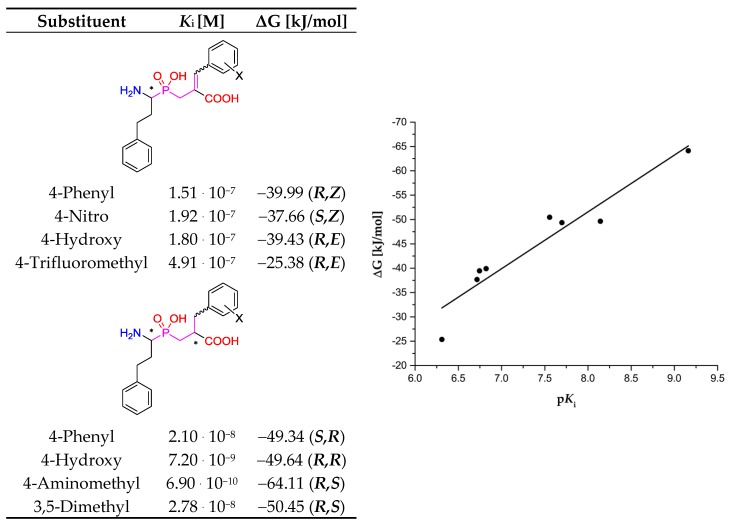
Correlation plot of inhibitory activity of phosphinic dipeptide and dehydrodipeptide analogs (p*K*_i_) reported for human aminopeptidase N [8,10] with the corresponding values of the calculated free Gibbs energy (R^2^ = 0.91).

The results of our screening can be considered as qualitative. Globally, there is no significant correlation between experimental affinities of the described inhibitors and the corresponding values of the computed free Gibbs energy. Too many variables influence the reported data based on experimental procedures (e.g., different protocols of inhibition measurements and calculations) and the data based on calculations (structural, stereochemical, and conformational implications, for both enzymes and ligands). Nevertheless, regressions that involved limited datasets showed clearer tendencies. For the first relationship, we selected the universal inhibitor (bestatin), which was assayed with several aminopeptidases, and prepared the plot of ΔG versus p*K*_i_ (Table 9). Although the correlation coefficient for the linear regression is not remarkable (R^2^ = 0.65), the trend is visible. Much more pronounced correlation (R^2^ = 0.91) was envisaged for the specified type of inhibitors (phosphinic (dehydro)dipeptide analogs) and a single aminopeptidase (human aminopeptidase N, M01.001, Figure 11). Although these compounds were tested as a diastereomeric mixture [8,10], while the binding energies were calculated for the best single diastereoisomers, the obtained fit to linear regression is remarkable. Thus, the applied procedure can be recommended for inhibitors of a particular structure.

## Figures and Tables

**Table 1:** Metallo-aminopeptidases included in the MEROPS database and selected for this study; the originating organisms and the crystal structures are referenced. PDB, Protein Data Bank.

MEROPS ID	Name	Organism	PDB Code	Metal Ion(s)
M01.001	Aminopeptidase N	*Homo sapiens*	4FYT [14]	Zn
M01.001	Aminopeptidase N	*Sus scrofa*	4FKE [15]	Zn
M01.003	Aminopeptidase A	*Homo sapiens*	4KX7 [16]	Zn
M01.004	Leukotriene A4 hydrolase	*Homo sapiens*	4MKT [17]	Zn
M01.005	Aminopeptidase N	*Escherichia coli*	3B34 [18]	Zn
M01.011	Cystinyl aminopeptidase	*Homo sapiens*	5MJ6 [19]	Zn
M01.021	Tricorn interacting factor F3	*Thermoplasma acidophilum*	1Z5H [20]	Zn
M01.024	ERAP2 aminopeptidase	*Homo sapiens*	5AB0 [21]	Zn
M01.029	M1 aminopeptidase Ey	*Plasmodium falciparum*	3EBH [22]	Zn
M01.031	Cold-active aminopeptidase	*Colwellia psychrerythraea*	3CIA [23]	Zn
M01.034	Leukotriene A4 hydrolase	*Saccharomyces cerevisiae*	2XQ0 [24]	Zn
M01.uns	Aminopeptidase N	*Neisseria meningitidis*	2GTQ [25]	Zn
M17.001	Leucyl aminopeptidase	*Bos taurus*	1LAM [26]	Zn, Zn
M17.002	Leucyl aminopeptidase	*Solanum lycopersicum*	4KSI [27]	Mg, Mg
M17.003	Aminopeptidase A	*Escherichia coli*	1GYT [28]	Zn, Zn
M17.016	Aminopeptidase A/I	*Helicobacter pylori*	4ZLA [29]	Zn, Zn
M17.A05	Leucyl aminopeptidase	*Caenorhabditis elegans*	2HC9 [30]	Zn, Zn
M17.uns	Leucyl aminopeptidase	*Pseudomonas putida*	3H8G [31]	Mn, Zn
M17.uns	Leucyl aminopeptidase	*Xanthomonas oryzae*	3JRU [32]	Zn, Zn
M18.002	Aspartyl aminopeptidase	*Bos taurus*	3VAT [33]	Mg, Zn
M18.002	Aspartyl aminopeptidase	*Homo sapiens*	4DYO [34]	Zn, Zn
M18.002	Aspartyl aminopeptidase	*Pseudomonas aeruginosa*	4NJR [35]	Zn, Zn
M18.003	Aspartyl aminopeptidase	*Plasmodium falciparum*	4EME [36]	Zn, Zn
M18.004	Aminopeptidase I	*Clostridium acetobutylicum*	2GLJ [37]	Mn, Mn
M18.004	Aminopeptidase I	*Thermotoga maritima*	2GLF [38]	Mn, Mn
M24.001	Methionyl aminopeptidase 1	*Escherichia coli*	2GGC [39]	Co, Co
M24.001	Methionyl aminopeptidase 1	*Mycobacterium tuberculosis*	1YJ3 [40]	Co, Co
M24.001	Methionyl aminopeptidase 1	*Rickettsia prowazekii*	3MX6 [41]	Mn, Mn
M24.002	Methionyl aminopeptidase 2	*Homo sapiens*	1B6A [42]	Co, Co
M24.017	Methionyl aminopeptidase 1	*Homo sapiens*	2GZ5 [43]	Co, Co
M24.035	Methionyl aminopeptidase	*Pyrococcus furiosus*	1XGS [44]	Co, Co
M24.036	Methionyl aminopeptidase 1	*Staphylococcus aureus*	1QXY [45]	Co, Co, Co
M24A.un	Methionyl aminopeptidase 2	*Encephalitozoon cuniculi*	3FM3 [46]	Fe, Fe
M24.004	Aminopeptidase P	*Escherichia coli*	1WL9 [47]	Mn, Mn
M24.009	Aminopeptidase P1	*Homo sapiens*	3CTZ [48]	Mn, Mn
M24.026	Aminopeptidase P3	*Homo sapiens*	5 × 49 [49]	Mn, Mn
M24.031	Aminopeptidase P	*Thermotoga maritima*	2ZSG [50]	Zn
M24.038	Aminopeptidase P	*Plasmodium falciparum*	5JR6 [51]	Mn, Mn
M24.A11	Aminopeptidase P	*Xanthomonas campestris*	5CDE [52]	Zn, Zn
M28.003	Aminopeptidase S	*Streptomyces griseus*	1CP7 [53]	Zn, Zn
M28.uns	Non-peptidase homologues	*Clostridium acetobutylicum*	3K9T [54]	Zn
M28.uns	Non-peptidase homologues	*Parabacteroides distasonis*	3TC8 [55]	Zn
M28.002	Aminopeptidase Ap1	*Vibrio proteolyticus*	1RTQ [56]	Zn, Zn
M28.020	AM-1 aminopeptidase	*Aneurinibacillus sp. AM-1*	2EK8 [57]	Zn, Zn
M29.001	Aminopeptidase T	*Thermus thermophilus*	2AYI [58]	Zn, Zn
M29.005	Aminopeptidase S	*Staphylococcus aureus*	1ZJC [59]	Co, Co
M42.001	Glutamyl aminopeptidase	*Bacillus subtilis*	1VHE [60]	Zn
M42.001	Glutamyl aminopeptidase	*Streptococcus pneumoniae*	3KL9 [61]	Zn, Zn
M42.003	PhTET1 aminopeptidase	*Pyrococcus horikoshii*	2CF4 [62]	Co, Co
M42.004	PhTET2 aminopeptidase	*Pyrococcus horikoshii*	1Y0Y [63]	Zn, Zn
M42.009	PhTET3 aminopeptidase	*Pyrococcus horikoshii*	2VPU [64]	Zn, Zn
M55.001	D-aminopeptidase DppA	*Bacillus subtilis*	1HI9 [65]	Zn, Zn

**Table 2:** Selected M1 aminopeptidases with the P1′-preferred amino acid in the S1′ binding pocket with residues forming the cavity.

Merops ID	Enzyme Name	P1′	Amino Acids of S1′
M01.001	Aminopeptidase N	F, G, W	Gly352, Ala353, Arg381, Thr384, Val385, His388, Glu389, Ser415, Glu418, Tyr477
M01.003	Aminopeptidase A	E, M, R	Gly357, Ala358, Asn371, Gln385, Thr389, Val390, His393, Glu394, Ser420, Glu423, Phe424, Asp444, Tyr479, Ser480
M01.004	Leukotriene A4 hydrolase	G, H	Gly268, Gly269, Asp291, Val292, His295, Glu296, Val322, Glu325, Tyr383, Arg563, Lys565
M01.005	Aminopeptidase N (bacterial)	G, N	Gly261, Ala262, Tyr275, Arg293, Val294, His297, Asp327, Tyr381, Glu382
M01.011	Cystinyl aminopeptidase	R, T, V	Gly428, Ala429, Thr442, Leu457, Lys460, Ile461, His464, Glu465, Thr491, Glu494, Tyr549
M01.024	ERAP2 aminopeptidase	T, Y, V	Gly334, Ala335, Ser348, Trp363, Arg366, Val376, His370, Glu371, Lys397, Glu400, Tyr455

**Table 3:** Selected members of the M1 family with preferred P1′ substituents (R, compare Figure 1) that were the most favored, had the best interaction with the catalytic metal ion via the phosphinate group, and were the most specific (compare explanation in the text) are shown. The stereochemistry of the ligands and the calculated Gibbs free energy of binding to the active center are included. Known inhibitors are given as the references, with inhibition potency (*K*_i_ or IC_50_) and calculated ΔG values.

Enzyme	Inhibitor	P1′ Substituent/Known Inhibitors	Configuration	ΔG [kJ/mol]
M01.001 *Homo sapiens* 4FYT	The best found	4-Hydroxy-3-methoxyphenyl	*S,S*	−53.82
	Metal-interacting			
	The most specific	5-Amino-thiophen-2-yl	*R,S*	−34.19
	*K*_i_ = 0.69 nM	R = 4-(Aminomethyl)phenyl [8]		−64.11
M01.001 *Sus scrofa* 4FKE	The best found	4-Boronophenyl MIDA ester	*R,R*	−39.09
	Metal-interacting			
	The most specific			
	IC_50_ = 8.10 µM	Bestatin [77]		−45.38
M01.003 *Homo sapiens* 4KX7	The best found	3-Isobutylaziridin-2-yl	*R,S*	−38.80
	Metal-interacting			
	The most specific	1-Methyl-1*H*-imidazol-5-yl	*R,S*	−26.34
	*K*_i_ = 75 µM	Bestatin [16]		−7.54
M01.004 *Homo sapiens* 4MKT	The best found	2-Borono-5-methoxyphenyl	*R,S*	−57.39
	Metal-interacting	none		
	The most specific	2-Butyl-5-chloro-1*H*-imidazol-4-yl	*R,S*	−40.94
	*K*_i_ = 200 nM	Bestatin [78]		−53.73
M01.005 *Escherichia coli* 3B34	The best found	3,4-Dihydroxyphenyl	*R,E*	−49.91
	Metal-interacting			
	The most specific	none		
	*K*_i_ = 1.5 nM	Ala-P[(O)(OH)CH_2_]-Phe-Phe [79]		−50.47
M01.011 *Homo sapiens* 5MJ6	The best found	3-(Furan-2-carbonyl)quinolin-2-yl	*R,S*	−117.58
	Metal-interacting			
	The most specific	none		
	*K*_i_ = 1.7 nM	IVDE77^1^ [80]		−53.58
M01.021 *Thermoplasma acidophilum* 1Z5H	The best found	4-(6-(Hydroxymethyl)pyridin-2-yl)phenyl	*R,S*	−37.43
	Metal-interacting	6-Ethyl-4-oxo-4*H*-chromen-3-yl	*R,S*	−37.15
	The most specific			−37.15
	Unknown	Bestatin		−6.42
M01.024 *Homo sapiens* 5AB0	The best found	2-Benzyloxyphenyl	*R,S*	−76.49
	Metal-interacting	Phenanthren-4-yl	*R,Z*	−36.15
	The most specific	Diphenylmethyl	*R,Z*	−52.41
	IC_50_ = 237 nM	DABA-Trp derivative^2^ [81]		−54.74
M01.029 *Plasmodium falciparum* 3EBH	The best found	3-Hydroxy-4-methoxyphenyl	*R,S*	−63.40
	Metal-interacting			
	The most specific	4-(2-Pyridyl)phenyl	*R,E*	−63.35
	IC_50_ = 6 nM	BDM14471^3^ [82]		−37.38
M01.031 *Colwellia psychrerythraea* 3CIA	The best found	2-(*tert*-Butylthio)phenyl	*R,R*	−76.28
	Metal-interacting			
	The most specific			
	Unknown value	Bestatin		−66.93
M01.034 *Saccharomyces cerevisiae* 2XQ0	The best found	3,5-Di-*tert*-butylphenyl	*S,R*	−53.62
	Metal-interacting			
	The most specific	3-phenyl-1*H*-pyrazol-4-yl	*R,R*	−44.73
	Unknown value	Bestatin		−47.06
M01.un *Neisseria meningitides* 2GTQ	The best found	Quinolin-4-yl	*R,E*	−54.98
	Metal-interacting			
	The most specific	Biphenyl-2-yl	*R,E*	−45.78
	*K*_i_ = 54 nM	R = 4-Hydroxyphenyl [83]		−26.61

^1^ IVDE77, peptidomimetic analogue β^2^hVal-Tyr-Ile-Aia-Nva-Phe; β^2^HVal, β^2^-homovaline; Aia, 4-amino-1,2,4,5-tetrahydro-indolo [2,3-*c*]azepin-3-on, a constrained Trp analogue. ^2^ (*S*)-2-(3-Amino-4-((*S*)-2-aminohexanamido)benzamido)-3-(1*H*-indol-3-yl)propanoic acid. ^3^ BDM14471, (2*Z*)-2-Benzylidene-*N*-(4-fluorobenzyl)-*N′*-hydroxymalonamide.

**Table 4:** *Homo sapiens* M18 and M24 aminopeptidases with the P1′-preferred amino acid in the S1′ binding pocket with residues forming the cavity.

Merops ID	Enzyme Name	P1′	Amino Acids of S1′
M18.002	Aspartyl aminopeptidase	A, H, L	Gly123, Glu301, Glu302, Val303, Gly304, Ser305, Gly414, Thr415, His440
M24.002	Methionyl aminopeptidase 2	A, S, V	Ala230, Asn327, Leu328, Asn329, Gly330, His331, His339, Thr343, Glu364, Phe366, Leu447
M24.017	Methionyl aminopeptidase 1	A, S, V	Cys211, Tyr300, Gly302, His303, His310, Val315, His317, Glu336, Met338, Gln365
M24.009	Xaa-Pro aminopeptidase 1	P	His485, His489, Glu523, Tyr527, Arg535, Glu537
M24.026	Xaa-Pro aminopeptidase 3	A, S, P	Asp110, Trp163, Leu313, His420, His421, Val422, Gly423, His424, His431, Glu451, Pro452, Arg473

**Table 5:** Selected human metallo-aminopeptidases with P1′ substituents that were the most favored, had the best interaction with the catalytic metal ion via the phosphinate group, and were the most specific are shown. The stereochemistry of the ligands and the calculated Gibbs free energy of binding in the active center are included. Known inhibitors are given as references.

Enzyme	Inhibitor	P1′ Substituent/Known Inhibitors	Configuration	ΔG [kJ/mol]
M18.002 *Homo sapiens* 4DYO	The best found	3,4-Dibenzyloxyphenyl	*R,S*	−88.05
	The most specific	None		
	Metal-interacting	5-(Ethoxymethyl)furan-2-yl	*S,S*	−46.49
	C = 50 μg/mL – 50%	Phenantroline [87]		−16.74
M24.002 *Homo sapiens* 1B6A	The best found	4-Borono-2,3-difluorophenyl	*R,S*	−84.15
	The most specific	3-(3-Chlorophenyl)propyl	*S,S*	−55.00
	Metal-interacting	Phenylpropyl	*S,S*	−59.33
	*K*_i_ = 26 nM	Beloranib [88]		−28.39
M24.017 *Homo sapiens* 2GZ5	The best found	4-Boronophenyl	*R,E*	−85.18
	The most specific	4-Borono-3-fluorophenyl	*R,R*	−51.19
	Metal-interacting	4-(6-(Hydroxymethyl)pyridinyl)phenyl	*S,R*	−77.96
	*K*_i_ = 10 nM	Barbiturate-based inhibitor^1^ [89]		−53.46
M24.009 *Homo sapiens* 3CTZ	The best found	4-Borono-2-fluorophenyl	*R,R*	−51.91
	The most specific	2-Boronophenyl	*S,S*	−41.61
	Metal-interacting	None		
	*K*_i_ = 260 nM	GW796406^2^ [90]		−42.11
M24.026 *Homo sapiens* 5 × 49	The best found	3-Phenylaziridin-2-yl	*R,S*	−102.22
	The most specific	2-Borono-6-fluorophenyl	*R,R*	−68.93
	Metal-interacting	3-Borono-5-isopropoxyphenyl	*R,R*	−83.35
	*K*_i_ = 640 nM	Apstatin [91]		−103.24

^1^ 5-[(*p*-Dimethylaminostyryl)methylidene]barbituric acid. ^2^ GW796406, *N*-[(2*S*)-2-(mercaptomethyl)-3-methylbutanoyl]-4-(1*H*-pyrazol-1-yl)-l-phenylalanine.

**Table 6:** Selected bacterial aminopeptidases with the P1′-preferred amino acid in the S1′ binding pocket with residues forming the cavity.

Merops ID	Enzyme Name	P1′	Amino Acids of S1′
M17.016	Aminopeptidase A/I	ND^1^	Lys258, Lys270, Asn338, Asp340, Ala341, Arg344. Leu368, Thr369, Gly370, Ala371, Ile428, Gly435, Thr438, Ala439
M24.036	Aminopeptidase S	ND^1^	Ala75, Asp104, Leu165, Thr166, His168, His175, Ala179, His180, Glu202, Phe204, Phe221
M42.001	Glutamyl aminopeptidase	ND^1^	Gly89, Gly90, Glu213, Glu214, Gly216, Leu217, Gly292, Thr293, His318

^1.^ The enzyme specificities with k_cat_ or K_M_ were not determined (ND).

**Table 7:** Selected bacterial metallo-aminopeptidases with P1′ substituents that were the most favored, had the best interaction with the catalytic metal ion via the phosphinate group, and were the most specific are shown. The stereochemistry of the ligands and the calculated Gibbs free energy of binding in the active center are included. Known inhibitors are given as references.

Enzyme	Inhibitor	P1′ Substituent/Known Inhibitors	Configuration	ΔG [kJ/mol]
M17.016 *Helicobacter pylori* 4ZLA	The best found	3-Borono-6-ethoxyphenyl	*S,Z*	−81.94
	Metal-interacting			
	The most specific	None		
	IC_50_ = 49 nM	Bestatin [96]		−26.60
M24.036 *Staphylococcus aureus* 1QXY	The best found	Benzo [1,3]dioxol-5-yl	*R,R*	−93.88
	Metal-interacting			
	The most specific	2-Methylindol-7-yl	*R,S*	−59.33
	IC_50_ = 7 µM	3-Amino-1-(cyclopropylamino) heptan-2-one [45]		−34.55
M42.001 *Streptococcus pneumoniae* 3KL9	The best found	2-Chloro-5-phenylpyridin-3-yl	*R,Z*	−67.27
	Metal-interacting			
	The most specific	4-(Difluoromethoxy)-3-hydroxyphenyl	*R,S*	−56.17
	*K*_i_ = 292 nM	Bestatin^1^ [97]		−41.97

^1^ Inhibition measured for the *Thermotoga maritima* tetrahedral-shaped dodecameric aminopeptidase of the M42 family.

**Table 8:** Selected ADMET properties for M1 predicted inhibitors: MW, molecular weight; H-acc, H-bond acceptor number; H-don, H-bond donor number; LogP, logarithm of partition coefficient; GI, gastrointestinal absorption; Lipinski, fulfilling the rule of five; otherwise, the exceptions are given in parentheses.

P1′ Group	MW	H-acc	H-don	LogP	Water Solubility	GI	Lipinski
4-Hydroxy-3-methoxyphenyl	406.39	6	2	0.5	Moderately soluble	Low	Yes
5-Amino-thiophen-2-yl	381.41	4	2	0.16	Soluble	Low	Yes
4-Boronophenyl MIDA ester	515.28	8	1	−1.61	Moderately soluble	Low	1 (MW)
3-Isobutylaziridin-2-yl	368.41	4	2	−1.13	Soluble	High	Yes
1-Methyl-1*H*-imidazol-5-yl	365.36	4	2	−0.64	Soluble	High	Yes
2-Borono-5-methoxyphenyl	434.21	7	3	−1.07	Soluble	Low	Yes
2-Butyl-5-chloro-1*H*-imidazol-4-yl	440.88	5	2	1.19	Poorly soluble	Low	Yes
3,4-Dihydroxyphenyl	390.35	6	3	0.12	Soluble	Low	Yes
3-(Furan-2-carbonyl)quinolin-2-yl	505.48	7	1	1.62	Poorly soluble	Low	1 (MW)
4-(6-(Hydroxymethyl)pyridin-2-yl)phenyl	467.47	6	2	1.02	Poorly soluble	Low	Yes
6-Ethyl-4-oxo-4*H*-chromen-3-yl	456.45	6	1	1.33	Poorly soluble	Low	Yes
2-Benzyloxyphenyl	466.49	5	1	2.1	Poorly soluble	High	Yes
Diphenylmethyl	448.47	4	1	2.34	Poorly soluble	High	Yes
Phenanthren-4-yl	458.47	4	1	2.67	Poorly soluble	High	Yes
3-Hydroxy-4-methoxyphenyl	406.39	6	2	0.38	Moderately soluble	Low	Yes
4-(2-Pyridyl)benzaldehyde	435.43	5	1	1.38	Poorly soluble	High	Yes
2-(*tert*-Butylthio)phenyl	448.54	4	1	2.09	Poorly soluble	Low	Yes
3,5-Di-*tert*-butylphenyl	472.58	4	1	3.28	Poorly soluble	High	Yes
3-Phenyl-1*H*-pyrazol-4-yl	426.43	5	2	0.77	Poorly soluble	Low	Yes
Quinolin-4-yl	409.39	5	1	1.04	Moderately soluble	High	Yes
Biphenyl-2-yl	434.44	4	1	2.27	Poorly soluble	High	Yes

**Table 9:** Correlation plot of inhibitory activity of bestatin (p*K*_i_) reported for different metallo-aminopeptidases with the corresponding values of the calculated free Gibbs energy (R^2^ = 0.65).

**Enzyme**	***K*_i_ [M]**	**ΔG [kJ/mol]**	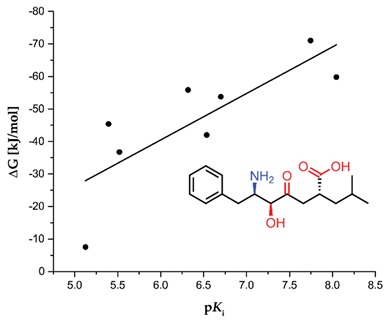
M01.001	3.03 · 10^−6^ [98]	−36.74	
M01.001	4.05 · 10^−6^ [77]	−45.38	
M01.003	7.50 · 10^−6^ [78]	−7.54	
M01.004	2.00 · 10^−7^ [79]	−53.73	
M01.029	4.81 · 10^−7^ [22]	−55.83	
M17.001	9.00 · 10^−9^ [98]	−59.77	
M28.002	1.80 · 10^−8^ [99]	−71.01	
M42.001	2.92 · 10^−7^ [97]	−41.97	

## Supplementary Materials

The following are available online at https://www.mdpi.com/2218-273X/10/4/659/s1, List of commercially available aldehydes considered in the study; Table S1. The full list of enzymes with preferred P1′ substituents that were the most favored, had the best interaction with the catalytic metal ion via the phosphinate group, and were the most specific; Table S2. Selected ADMET properties for the best inhibitors; Figure S1. Modeled binding mode of a phosphinic dipeptide analog containing a 5-amino-thiophen-2-yl P1′ fragment in the active center of human aminopeptidase N (PDB: 4FYT); Figure S2. Modeled binding mode of a phosphinic dipeptide analog containing a quinolin-4-yl P1′ fragment in the active center of *Neisseria meningitides* aminopeptidase (PDB: 2GTQ); Figure S3. Modeled binding mode of a phosphinic dipeptide analog containing quinolin-4-yl (carbon atoms are colored light green) and biphenyl-2-yl (carbon atoms are colored gray) P1′ fragments in the active center of *Neisseria meningitides* aminopeptidase (PDB: 2GTQ); Figure S4. Modeled binding mode of a phosphinic dipeptide analog containing a 2-benzyloxyphenyl P1′ fragment in the active center of ERAP2 aminopeptidase (PDB: 5AB0); Figure S5. Modeled binding mode of a phosphinic dehydrodipeptide analog containing a diphenylmethyl P1′ fragment in the active center of ERAP2 aminopeptidase (PDB: 5AB0); Figure S6. Modeled binding mode of a phosphinic dipeptide analog containing a 2-*tert*-butylthiophenyl P1′ fragment in the active center of cold-active aminopeptidase (PDB: 5AB0); Figure S7. Modeled binding mode of a phosphinic dipeptide analog containing a 3-isobutylaziridin-2-yl P1′ fragment in the active center of aminopeptidase A (PDB: 4KX7); Figure S8. Modeled binding mode of a phosphinic dipeptide analog containing a 3,4-dibenzyloxyphenyl P1′ fragment in the active center of aspartyl aminopeptidase (PDB: 4DYO); Figure S9. Modeled binding mode of a phosphinic dipeptide analog containing a 3-phenylaziridin-2-yl P1′ fragment in the active center of aminopeptidase P3 (PDB: 5 × 49); Figure S10. Modeled binding mode of a phosphinic dipeptide analog containing a 4-borono-2,3-difluorophenyl P1′ fragment in the active center of methionyl aminopeptidase 2 (PDB: 1B6A).
