# Supplementary material for: P1′ Residue-Oriented Virtual Screening for Potent and Selective Phosphinic (Dehydro) Dipeptide Inhibitors of Metallo-Aminopeptidases
Source: Biomolecules. 2020 Apr 24;10(4):659. doi: 10.3390/biom10040659 (PMC7225938; doi:10.3390/biom10040659)
Supplement: Supplementary file 1 [file biomolecules-10-00659-s001.pdf]

biomolecules

Supplementary materials for

P1′ residue-oriented virtual screening for potent and selective phosphinic (dehydro)dipeptide inhibitors of metallo-aminopeptidases

Michal Talma[1] and Artur Mucha[1,*]

[1] Department of Bioorganic Chemistry, Wrocław University of Science and Technology, Wybrzeże Wyspiańskiego 27, 50-370 Wrocław, Poland; michal.talma@pwr.edu.pl, artur.mucha@pwr.edu.pl
* Correspondence: artur.mucha@pwr.edu.pl; Tel.: +48-71-320-3446 (A.M.)

Contents:

**S1. List of commercially available aldehydes considered in the study**	**3**
**S2. Full list of enzymes and the best inhibitors**	**11**
Table S1. The full list of enzymes with preferred P1′ substituents that were the most favored, had the best interaction with the catalytic metal ion via the phosphinate group, and were the most specific	11
**S3. ADMET properties of the best selected inhibitors from the Table S1**	**16**
Table S2. Selected ADMET properties for the best inhibitors	16
**S4. Additional figures mentioned in the main text**	**21**
Figure S1. Modeled binding mode of a phosphinic dipeptide analog containing a 5-amino-thiophen-2-yl P1′ fragment in the active center of human aminopeptidase N (PDB: 4FYT)	21
Figure S2. Modeled binding mode of a phosphinic dipeptide analog containing a quinolin-4-yl P1′ fragment in the active center of Neisseria meningitides aminopeptidase (PDB: 2GTQ)	22
Figure S3. Modeled binding mode of a phosphinic dipeptide analog containing quinolin-4-yl (carbon atoms are colored light green) and biphenyl-2-yl (carbon atoms are colored gray) P1′ fragments in the active center of Neisseria meningitides aminopeptidase (PDB: 2GTQ)	23
Figure S4. Modeled binding mode of a phosphinic dipeptide analog containing a 2-benzyloxyphenyl P1′ fragment in the active center of ERAP2 aminopeptidase (PDB: 5AB0)	24
Figure S5. Modeled binding mode of a phosphinic dehydrodipeptide analog containing a diphenylmethyl P1′ fragment in the active center of ERAP2 aminopeptidase (PDB: 5AB0)	25
Figure S6. Modeled binding mode of a phosphinic dipeptide analog containing a 2-tert-butylthiophenyl P1′ fragment in the active center of cold-active aminopeptidase (PDB: 5AB0)	26
Figure S7. Modeled binding mode of a phosphinic dipeptide analog containing a 3-isobutylaziridin-2-yl P1′ fragment in the active center of aminopeptidase A (PDB: 4KX7)	27

Figure S8. Modeled binding mode of a phosphinic dipeptide analog containing a 3,4-dibenzyloxyphenyl P1′ fragment in the active center of aspartyl aminopeptidase (PDB: 4DYO) 28
Figure S9. Modeled binding mode of a phosphinic dipeptide analog containing a 3-phenylaziridin-2-yl P1′ fragment in the active center of aminopeptidase P3 (PDB: 5X49) 29
Figure S10. Modeled binding mode of a phosphinic dipeptide analog containing a 4-borono-2,3-difluorophenyl P1′ fragment in the active center of methionyl aminopeptidase 2 (PDB: 1B6A) 30
S5. References **31**

S1. List of commercially available aldehydes considered in the study

Formaldehyde, acetaldehyde, trifluoroacetaldehyde, tribromoacetaldehyde, sodium glyoxylate, chloroacetaldehyde, 2-bromoacrolein, methylglyoxal, propionaldehyde, glyceraldehyde, 2,4-dichlorothiazole-5-carboxyaldehyde, heptafluorobutyraldehyde, 4-bromothiazole-2-carboxyaldehyde, 2-thazolecarboxaldehyde, 5-thiazolecarboxyaldehyde, 4-oxazolecarboxyaldehyde, 2-imidazolecarboxyaldehyde, 4-imidazolecarboxyaldehyde, 2-aminothiazole-5-carboxaldehyde, crotonaldehyde, cyclopropanecarboxaldehyde, methacrolein, butyraldehyde, isobutyraldehyde, 3-(methylthio)propionaldehyde, 3-methoxy-propionaldehyde, aldol aldrich, 2,2-dimethoxyacetaldehyde, 3,4-dibromothiophene-2-carboxaldehyde, 4-bromothiophene-2-carboxaldehyde, 3-bromothiophene-2-carboxaldehyde, 5-bromo-2-thiophenecarboxaldehyde, 4,6-dichloropyrimidine-5-carboxaldehyde, 2-amino-4,6-dichloropyrimidine-5-carboxaldehyde, 4-bromo-2-furaldehyde, 5-bromo-2-furaldehyde, 5-chloro-2-furaldehyde, 5-chloro-2-thiophenecarboxaldehyde, 5-nitro-2-thiophenecarboxaldehyde, 5-nitro-2-furaldehyde, 5-formyluracil, 2-thiophenecarboxaldehyde, 3-furancarboxaldehyde, 3-furancarboxaldehyde, furfural, 2-formyl-3-thiopheneboronic acid, 3-formyl-2-thienylboronic acid, 5-formyl-2-thienylboronic acid, 5-formyl-2-furanylboronic acid, 4-bromo-1-methyl-1H-imidazole-5-carboxaldehyde, pyrrole-2-carboxaldehyde, 4-methyl-2-thiazolecarboxaldehyde, 2-methylthiazole-4-carboxaldehyde, 4-methylthiazole-5-carboxaldehyde, 2-methyloxazole-4-carboxaldehyde, 5-methylisoxazole-3-carboxaldehyde, 2-aminopyrimidine-5-carboxaldehyde, 1-methyl-1H-pyrazole-4-carboxaldehyde, 1-methyl-2-imidazolecarboxaldehyde, 1-methyl-5-imidazolecarboxaldehyde, 4-methyl-1H-imidazole-2-carboxaldehyde, 4-methyl-5-imidazolecarboxaldehyde, 3-methylcrotonaldehyde, 4-pentenal, *trans*-2-pentenal, tetrahydrofuran-3-carboxaldehyde, methyl 4-oxobutanoate, 3-dimethylaminoacrolein, 2-methylbutyraldehyde, isovaleraldehyde, trimethylacetaldehyde, valeraldehyde, 3,5-dibromo-4-pyridinecarboxaldehyde, 3,5-dibromopyridine-2-carboxaldehyde, 2-chloro-4-iodopyridine-3-carboxaldehyde, 2-amino-4-chloro-5-formyl-3-thiophenecarbonitrile, 2,4-dichloropyridine-3-carboxaldehyde, 2,6-dichloro-4-pyridinecarboxaldehyde, 2,6-dichloropyridine-3-carboxaldehyde, 3,5-dichloro-4-pyridinecarboxaldehyde, 2-bromo-3-pyridinecarboxaldehyde, 2-bromo-4-pyridinecarboxaldehyde, 5-bromo-2-pyridinecarboxaldehyde, 3-bromo-4-pyridinecarboxaldehyde, 5-bromo-3-pyridinecarboxaldehyde, 6-bromo-2-pyridinecarboxaldehyde, 6-bromo-3-pyridinecarboxaldehyde, 2-chloro-3-pyridinecarboxaldehyde, 2-chloro-4-pyridinecarboxaldehyde, 3-chloro-4-pyridinecarboxaldehyde, 5-chloropyridine-2-carboxaldehyde, 6-chloropyridine-3-carboxaldehyde, 2-fluoro-3-pyridinecarboxaldehyde, 3-fluoro-4-pyridinecarbaldehyde, 4-bromo-3-methylthiophene-2-carboxaldehyde, 2-pyridinecarboxaldehyde, 3-pyridinecarboxaldehyde, 4-pyridinecarboxaldehyde, 2-amino-3-pyridinecarboxaldehyde, 4-methylthiophene-2-carboxaldehyde, 5-methyl-2-thiophenecarboxaldehyde, 5-methylfurfural, 5-hydroxymethyl-2-furaldehyde, N-methyl-2-pyrrolecarboxaldehyde, 2,4-dimethylthiazole-5-carboxaldehyde, 4,5-dimethylthiazole-2-carboxaldehyde, 3-(N-formyl-N-methylamino)-2-(trifluoromethyl)propionic acid, *trans,trans*-2,4-hexadienal, 2-methyl-2-pentenal, *trans*-2-hexen-1-al, cyclopentanecarboxaldehyde, *(R)*-(+)-2,2-dimethyl-1,3-dioxolane-4-carboxaldehyde, 2,2-dimethylbutanal, 2-ethylbutyraldehyde, 2-methylpentanal, 3,3-dimethylbutyraldehyde, hexanal,

2,3,5,6-tetrafluorobenzaldehyde, 4-bromo-2,6-difluorobenzaldehyde, 3,6-dibromo-2-fluorobenzaldehyde, 2,4,6-tribromo-3-hydroxybenzaldehyde, 2-chloro-3,6-difluorobenzaldehyde, 3-chloro-2,6-difluorobenzaldehyde, 4-chloro-2,6-difluorobenzaldehyde, 2,3,6-trichlorobenzaldehyde, 2,3,5-trifluorobenzaldehyde, 3,4,5-trifluorobenzaldehyde, 2,4,5-trifluorobenzaldehyde, 2-bromo-4-chlorobenzaldehyde, 3-bromo-5-chlorobenzaldehyde, 3-bromo-5-chloro-4-hydroxybenzaldehyde, 3-bromo-5-chlorosalicylaldehyde, 2-bromo-5-fluorobenzaldehyde, 2-bromo-6-fluorobenzaldehyde, 3-bromo-4-fluorobenzaldehyde, 4-bromo-3-fluorobenzaldehyde, 4-bromo-2-fluorobenzaldehyde, 5-bromo-2-fluorobenzaldehyde, 5-bromo-3-fluorobenzaldehyde, 5-bromo-3-fluorosalicylaldehyde, 3-bromo-5-nitrobenzaldehyde, 4-bromo-5-nitrobenzaldehyde, 3-bromo-5-nitrosalicylaldehyde, 5-bromo-3-nitrosalicylaldehyde, 2,5-dibromobenzaldehyde, 3,5-dibromobenzaldehyde, 3,5-dibromo-4-hydroxybenzaldehyde, 2,5-dibromosalicylaldehyde, 2-chloro-4-flurobenzaldehyde, 2-chloro-6-flurobenzaldehyde, 3-chloro-2-flurobenzaldehyde, 4-chloro-3-flurobenzaldehyde, 3-chloro-5-fluro-4-hydroxybenzaldehyde, 3-chloro-5-flurosalicylaldehyde, 5-chloro-2-hydroxy-3-iodobenzaldehyde, 2-chloro-5-nitrobenzaldehyde, 2-chloro-6-nitrobenzaldehyde, 4-chloro-2-nitrobenzaldehyde, 4-chloro-3-nitrobenzaldehyde, 5-chloro-2-nitrobenzaldehyde, 2,3-dichlorobenzaldehyde, 2,4-dichlorobenzaldehyde, 2,5-dichlorobenzaldehyde, 2,6-dichlorobenzaldehyde, 3,4-dichlorobenzaldehyde, 3,5-dichlorobenzaldehyde, 2,4-dichloro-6-hydroxybenzaldehyde, 3,5-dichlorosalicylbenzaldehyde, 2-fluoro-5-iodobenzaldehyde, 2-fluoro-6-iodobenzaldehyde, 2-fluoro-5-nitrobenzaldehyde, 4-fluoro-3-nitrobenzaldehyde, 2,3-difluorobenzaldehyde, 2,4-difluorobenzaldehyde, 2,5-difluorobenzaldehyde, 2,6-difluorobenzaldehyde, 3,4-difluorobenzaldehyde, 3,5-difluorobenzaldehyde, 3,5-difluorosalicylaldehyde, 3,5-diiodosalicylaldehyde, 3,5-dinitrosalicylaldehyde, 2,4-dinitrobenzaldehyde, 2,6-dinitrobenzaldehyde, 6-(trifluoromethyl)pyridine-3-carboxyaldehyde, 2,3-difluoro-4-formylphenylboronic acid, 2,6-difluoro-3-formylphenylboronic acid, 2,4-difluoro-3-formylphenylboronic acid, 3,5-difluoro-4-formylphenylboronic acid, 2-bromobenzaldehyde, 3-bromobenzaldehyde, 4-bromobenzaldehyde, 2-bromo-3-hydroxybenzaldehyde, 2-bromo-5-hydroxybenzaldehyde, 3-bromo-2-hydroxybenzaldehyde, 3-bromo-4-hydroxybenzaldehyde, 4-bromo-2-hydroxybenzaldehyde, 5-bromosalicylaldehyde, 2-amino-3,5-dibromobenzaldehyde, 2-chlorobenzaldehyde, 3-chlorobenzaldehyde, 4-chlorobenzaldehyde, 2-chloro-3-hydroxybenzaldehyde, 2-chloro-4-hydroxybenzaldehyde, 2-chloro-6-hydroxybenzaldehyde, 3-chloro-2-hydroxybenzaldehyde, 3-chloro-4-hydroxybenzaldehyde, 5-chlorosalicylaldehyde, 2-fluorobenzaldehyde, 3-fluorobenzaldehyde, 4-fluorobenzaldehyde, 5-fluorosalicylaldehyde, 3-fluorosalicylaldehyde, 2-amino-5-(trifluoromethyl)nicotinaldehyde, 2-iodobenzaldehyde, 3-iodobenzaldehyde, 4-iodobenzaldehyde, 5-iodosalicylaldehyd, 2-nitrobenzaldehyde, 3-nitrobenzaldehyde, 4-nitrobenzaldehyde, 2-hydroxy-3-nitrobenzaldehyde, 2-hydroxy-5-nitrobenzaldehyde, 3-hydroxy-4-nitrobenzaldehyde, 4-hydroxy-3-nitrobenzaldehyde, 5-hydroxy-2-nitrobenzaldehyde, 2-fluoro-3-formylphenylboronic acid, 2-fluoro-4-formylphenylboronic acid, 3-fluoro-2-formylphenylboronic acid, 3-fluoro-4-formylphenylboronic acid, 4-fluoro-3-formylphenylboronic acid, 5-bromo-2-methoxypyridine-3-carboxaldehyde, 6-chloro-2-methoxypyridine-3-carboxaldehyde, benzaldehyde, 3-hydroxybenzaldehyde, 4-hydroxybenzaldehyde, salicylaldehyde, *trans*-3-(2-furyl)acrolein, 2,4-dihydroxybenzaldehyde, 2,3-dihydroxybenzaldehyde, 2,5-dihydroxybenzaldehyde, 3,4-dihydroxybenzaldehyde, 3,5-

dihydroxybenzaldehyde, 2,3,4-trihydroxybenzaldehyde, 2,4,5-trihydroxybenzaldehyde, 2,4,6-trihydroxybenzaldehyde, 3,4,5-trihydroxybenzaldehyde, 2-formylphenylboronic acid, 3-formylphenylboronic acid, 4-formylphenylboronic acid, 2-aminobenzaldehyde, 2-methylpyridine-3-carboxaldehyde, 6-methylpyridine-2-carboxaldehyde, 3-methylpyridine-2-carboxaldehyde, 6-methylpyridine-3-carboxaldehyde, 2-methoxy-3-pyridinecarboxaldehyde, 6-methoxy-2-pyridinecarboxaldehyde, 6-methoxy-3-pyridinecarboxaldehyde, 5-methoxy-3-pyridinecarboxaldehyde, 4,5-dimethylthiophene-2-carboxaldehyde, 5-ethyl-2-thiophenecarboxaldehyde, 4,5-dimethyl-2-furaldehyde, 5-ethyl-2-furaldehyde, 3,5-dimethylpyrrole-2-carboxaldehyde, 1-cyclohexene-1-carboxaldehyde, 3-cyclohexene-1-carboxaldehyde, cyclohexanecarboxaldehyde, 2-chloro-6-methylbenzaldehyde, 2,4-heptadienal, ethyl 2-formyl-1-cyclopropanecarboxylate, ethyl 3-methyl-4-oxocrotonate, 2,2-dimethyl-4-pentenal, *cis*-4-hepten-1-al, *trans*-2-heptenal, heptenaldehyde, 2,5-dimethoxy-3-tetrahydrofurancarboxaldehyde, N-boc-2-aminoacetaldehyde, 3,5-difluoro-4-formylbenzonitrile, 2-bromo-5-(trifluoromethyl)benzaldehyde, 3-bromo-5-(trifluoromethyl)benzaldehyde, 4-bromo-2-cyanobenzaldehyde, 2-chloro-3-(trifluoromethyl)benzaldehyde, 2-chloro-5-(trifluoromethyl)benzaldehyde, 3-chloro-5-(trifluoromethyl)benzaldehyde, 4-chloro-3-(trifluoromethyl)benzaldehyde, 5-chloro-2-(trifluoromethyl)benzaldehyde, 2-fluoro-5-formylbenzonitrile, 4-cyano-3-fluorobenzaldehyde, 2,2-difluoro-5-formylbenzodioxole, 2-nitro-4-(trifluoromethyl)benzaldehyde, 2-fluoro-4-(trifluoromethyl)benzaldehyde, 2-fluoro-3-(trifluoromethyl)benzaldehyde, 2-fluoro-5-(trifluoromethyl)benzaldehyde, 2-fluoro-6-(trifluoromethyl)benzaldehyde, 4-fluoro-2-(trifluoromethyl)benzaldehyde, 4-fluoro-3-(trifluoromethyl)benzaldehyde, 3-fluoro-5-(trifluoromethyl)benzaldehyde, 3-bromo-1h-indazole-5-carboxaldehyde, 6-bromo-1,3-benzodioxole-5-carboxaldehyde, 5-bromo-1,3-benzodioxole-4-carboxaldehyde, 3-chloro-1H-indazole-5-carboxaldehyde, 2-(trifluoromethyl)benzaldehyde, 3-(trifluoromethyl)benzaldehyde, 4-(trifluoromethyl)benzaldehyde, 4-(trifluoromethylthio)benzaldehyde, 4-(trifluoromethoxy)benzaldehyde, 2-(trifluoromethoxy)benzaldehyde, 3-(trifluoromethoxy)benzaldehyde, 2-hydroxy-5-(trifluoromethoxy)benzaldehyde, 2-cyanobenzaldehyde, 3-formylbenzonitrile, 4-formylbenzonitrile, benzothiazole-2-carboxaldehyde, 6-nitropiperonal, 2-bromo-3-chloro-4-hydroxy-5-methoxybenzaldehyde, 3,5-dibromo-2-methoxybenzaldehyde, 3,5-dibromo-2-hydroxy-4-methoxybenzaldehyde, 6-chloro-2-fluoro-3-methylbenzaldehyde, 2,6-difluoro-4-methoxybenzaldehyde, 4-(difluoromethoxy)benzaldehyde, 2-(difluoromethoxy)benzaldehyde, 4-(difluoromethoxy)-3-hydroxybenzaldehyde, 1H-indazole-6-carboxaldehyde, indazole-5-carboxaldehyde, 7-azaindole-3-carboxaldehyde, 4-(1H-tetraazol-5-yl)benzaldehyde, phenylglyoxal, 2,3-(methylenedioxy)benzaldehyde, piperonal, 3-formyl-4-hydroxybenzoic acid, 4-formyl-3-hydroxybenzoic acid, 5-formylsalicylic acid, 2-formyl-4,5-methylenedioxyphenylboronic acid, 2-bromo-4-methylbenzaldehyde, 3-bromo-4-methylbenzaldehyde, 3-bromo-4-methoxybenzaldehyde, 2-bromo-4-methoxybenzaldehyde, 2-bromo-5-methoxybenzaldehyde, 4-bromo-2-methoxybenzaldehyde, 5-bromo-2-methoxybenzaldehyde, 2-bromo-3-hydroxy-4-methoxybenzaldehyde, 5-bromo-2-hydroxy-3-methoxybenzaldehyde, 5-bromovanillin, 6-bromo-2-hydroxy-3-methoxybenzaldehyde, 2-chloro-4-methylbenzaldehyde, 2-chloro-6-methylbenzaldehyde, 3-chloro-4-methylbenzaldehyde, 4-chloro-2-methylbenzaldehyde, 4-chloro-3-methylbenzaldehyde, 2-chloro-3-methoxybenzaldehyde, 3-chloro-

4-methoxybenzaldehyde, 2-chloro-3-hydroxy-4-methoxybenzaldehyde, 5-chloro-2-hydroxy-4-methyl-benzaldehyde, 3-chloro-4-hydroxy-5-methoxybenzaldehyde, 5-chloro-2-hydroxy-3-methoxybenzaldehyde, 2-fluoro-6-methylbenzaldehyde, 3-fluoro-2-methylbenzaldehyde, 3-fluoro-4-methylbenzaldehyde, 4-fluoro-2-methylbenzaldehyde, 4-fluoro-3-methylbenzaldehyde, 5-fluoro-2-methylbenzaldehyde, 2-fluoro-5-methoxybenzaldehyde, 2-fluoro-3-methoxybenzaldehyde, 2-fluoro-4-methoxybenzaldehyde, 2-fluoro-6-methoxybenzaldehyde, 3-fluoro-4-methoxybenzaldehyde, 4-fluoro-3-methoxybenzaldehyde, 5-fluoro-2-methoxybenzaldehyde, 5-fluoro-3-methylsalicylaldehyde, 3-fluoro-4-hydroxy-5-methoxybenzaldehyde, 3-iodo-4-methoxybenzaldehyde, 5-iodo-2-methoxybenzaldehyde, 3-hydroxy-2-iodo-4-methoxybenzaldehyde, 5-iodovanillin, 4-methyl-3-nitrobenzaldehyde, 2-methoxy-4-nitrobenzaldehyde, 2-methoxy-5-nitrobenzaldehyde, 3-methoxy-2-nitrobenzaldehyde, 4-methoxy-3-nitrobenzaldehyde, 2-hydroxy-5-methoxy-3-nitrobenzaldehyde, 3-methoxy-5-nitrosalicylaldehyde, 5-nitrovanillin, 3-bromo-5-formyl-2-methoxyphenylboronic acid, 5-fluoro-3-formyl-2-methoxyphenylboronic acid, *m*-tolualdehyde, *o*-tolualdehyde, *p*-tolualdehyde, phenylacetaldehyde, 2-(methylthio)benzaldehyde, 4-(methylthio)benzaldehyde, 2-hydroxy-3-methylbenzaldehyde, 2-hydroxy-5-methylbenzaldehyde, 2-methoxybenzaldehyde, 4-hydroxy-3-methylbenzaldehyde, m-anisaldehyde, p-anisaldehyde, 2,4-dihydroxy-6-methylbenzaldehyde, 2-hydroxy-4-methoxybenzaldehyde, 2-hydroxy-5-methoxybenzaldehyde, 2-hydroxy-6-methoxybenzaldehyde, 3-hydroxy-4-methoxybenzaldehyde, 4-hydroxy-2-methoxybenzaldehyde, vanillin, *o*-vanillin, 2,4-dihydroxy-5-methoxybenzaldehyde, 3,4-dihydroxy-5-methoxybenzaldehyde, 5-acetoxymethyl-2-furaldehyde, 3-formyl-5-methylphenylboronic acid, 2-formyl-4-methoxyphenylboronic acid, 3-formyl-2-methoxyphenylboronic acid, 3-formyl-4-methoxyphenylboronic acid, 2,6-dimethoxypyridine-3-carboxaldehyde, ethyl 4-formylpyrrole-2-carboxylate, pyridoxal, bicyclo[2.2.1]hept-5-ene-2-carboxaldehyde, bicyclo[2.2.1]heptane-2-carboxaldehyde, 5-(ethoxymethyl)furan-2-carboxaldehyde, 2-butyl-5-chloro-1h-imidazole-4-carboxaldehyde, 2-octynal, 2-ethyl-2-hexenal, *trans*-2-octenal, 2-ethylhexanal, octanal, boc-l-alaninal, aldehyde name, 2,5-bis(trifluoromethyl)benzaldehyde, 3,5-bis(trifluoromethyl)benzaldehyde, 3-bromobenzothiophene-2-carboxaldehyde, 5′-bromo-2,2′-bithiophene-5-carboxaldehyde, 5-bromoindole-3-carboxaldehyde, 6-bromoindole-3-carboxaldehyde, 5-chloroindole-3-carboxaldehyde, 5-fluoroindole-3-carboxaldehyde, 2,6-difluorocinnamic aldehyde, 3-(1,1,2,2-tetrafluoroethoxy)benzaldehyde, 4-(1,1,2,2-tetrafluoroethoxy)benzaldehyde, 7-iodoindole-3-carboxaldehyde, 4-nitroindole-3-carboxaldehyde, 2-ethynylbenzaldehyde, 3-ethynylbenzaldehyde, 4-ethynylbenzaldehyde, phenylpropargyl aldehyde, benzo[b]thiophene-2-carboxaldehyde, thianaphthene-3-carboxaldehyde, 2,2′-bithiophene-5-carboxaldehyde, 3,3′-bithiophene-5-carboxaldehyde, 2-benzofurancarboxaldehyde, α-bromocinnamaldehyde, *trans*-4-bromocinnamaldehyde, 4-chlorocinnamaldehyde, 4-fluorocinnamaldehyde, α-chlorocinnamaldehyde, (2-bromo-4-formylphenoxy)acetic acid, (4-chloro-2-formylphenoxy)acetic acid, 2-methyl-3-(trifluoromethyl)benzaldehyde, 4-methyl-3-(trifluoromethyl)benzaldehyde, 5-methyl-2-(trifluoromethyl)benzaldehyde, 2-methoxy-4-(trifluoromethoxy)benzaldehyde, indole-2-carboxaldehyde, indole-3-carboxaldehyde, indole-4-carboxaldehyde, indole-5-carboxaldehyde, indole-6-carboxaldehyde, indole-7-carboxaldehyde, 5-cyano-2-methoxybenzaldehyde, 4-nitrocinnamaldehyde, *trans*-2-nitrocinnamaldehyde, methyl 3-formyl-4-nitrobenzoate, methyl 4-

formyl-3-nitrobenzoate, 2-(2-formyl-4-nitrophenyl)sulfanylacetic acid, 2-bromo-3-chloro-5-ethoxy-4-hydroxybenzaldehyde, *trans*-cinnamaldehyde, 3-vinylbenzaldehyde, 2,3-dihydrobenzofuran-5-carboxaldehyde, 3-(2-hydroxyphenyl)-2-propenal, 2-acetylbenzaldehyde, 3-acetylbenzaldehyde, 4-acetylbenzaldehyde, 1,4-benzodioxan-6-carboxaldehyde, 4-acetoxybenzaldehyde, methyl 3-formylbenzoate, methyl 4-formylbenzoate, 2-formylphenoxyacetic acid, 4-formylphenoxyacetic acid, methyl 3-formyl-4-hydroxybenzoate, 4-bromo-3,5-dimethoxybenzaldehyde, 5-bromo-2,3-dimethoxybenzaldehyde, 5-bromo-2,4-dimethoxybenzaldehyde, 5-bromoveratraldehyde, 6-bromoveratraldehyde, 3-(3-chlorophenyl)propionaldehyde, 2-chloro-3,4-dimethoxybenzaldehyde, 3-chloro-4,5-dimethoxybenzaldehyde, 5-chloro-2,3-dimethoxybenzaldehyde, 3-iodo-4,5-dimethoxybenzaldehyde, 5-iodo-2,4-dimethoxybenzaldehyde, 3-chloro-6-hydroxy-2,4-dimethyl-benzaldehyde, 2-iodo-4,5-dimethylbenzaldehyde, indoline-7-carboxaldehyde, 2-(3-formylphenoxy)acetamide, 4-acetamidobenzaldehyde, 4-ethyl-3-nitrobenzaldehyde, 4-ethoxy-3-nitrobenzaldehyde, 6-nitroveratraldehyde, 4-dimethylamino-2-nitrobenzaldehyde, 2,4-dimethylbenzaldehyde, 2,5-dimethylbenzaldehyde, 2,6-dimethylbenzaldehyde, 2-ethylbenzaldehyde, 2-phenylpropionaldehyde, 3,4-dimethylbenzaldehyde, 3,5-dimethylbenzaldehyde, 4-ethylbenzaldehyde, hydrocinnamaldehyde, 2-ethoxybenzaldehyde, 3-ethoxybenzaldehyde, 4-ethoxybenzaldehyde, 4-hydroxy-3,5-dimethylbenzaldehyde, 4-methoxy-2-methylbenzaldehyde, 4-methoxy-3-methylbenzaldehyde, benzyloxyacetaldehyde, 2,3-dimethoxybenzaldehyde, 2,4-dimethoxybenzaldehyde, 2,5-dimethoxybenzaldehyde, 2,6-dimethoxybenzaldehyde, 2-(2-hydroxyethoxy)benzaldehyde, 3,4-dimethoxybenzaldehyde, 3,5-dimethoxybenzaldehyde, 3-(2-hydroxyethoxy)benzaldehyde, 3-(methoxymethoxy)benzaldehyde, 3-ethoxy-4-hydroxybenzaldehyde, 3-ethoxysalicylaldehyde, 2,6-dimethoxy-4-hydroxybenzaldehyde, 3,4-dimethoxy-5-hydroxybenzaldehyde, 4,6-dimethoxysalicylaldehyde, syringaldehyde, 3-ethoxy-5-formylphenylboronic acid, 3-formyl-2-methoxy-5-methylphenylboronic acid, 4-ethoxy-3-formylphenylboronic acid, 4-(dimethylamino)benzaldehyde, 1-(2-tetrahydropyranyl)-1h-pyrazole-5-carboxaldehyde, *trans*,*trans*-2,4-nonadienal, *trans*-2,*cis*-6-nonadienal, *(R)*-1,4-dioxaspiro[4.5]decane-2-carboxaldehyde, 4-ethyl-4-formylhexanenitrile, 2,6-dimethyl-5-heptenal, *trans*-2-nonenal, nonenal, 3,5,5-trimethylhexanal, 3-cyclohexylpropionaldehyde, 3-phenylaziridine-2-carboxaldehyde, aldehyde name, 6-bromo-4-chloro-3-formylcoumarin, 6,8-dibromo-3-formylchromone, 6-bromo-3-formylchromone, 7-bromochromone-3-carboxaldehyde, 4-chloro-3-formylcoumarin, 7-fluorochromone-3-carboxaldehyde, 6-fluorochromone-3-carboxaldehyde, 3-formyl-6-nitrochromone, 4-chloro-6-fluoro-2H-benzopyran-3-carboxaldehyde, 2-chloro-3-quinolinecarboxaldehyde, 4,6-dichloro-2H-benzopyran-3-carboxaldehyde, 5-(4-chlorophenyl)isoxazole-3-carboxaldehyde, 5-(4-fluorophenyl)isoxazole-3-carboxaldehyde, 3,5-dichloro-4-(prop-2-yn-1-yloxy)benzaldehyde, 3-formyl-1H-indole-5-carbonitrile, 3-formylchromone, 3-(4-Fluorophenyl)-1H-pyrazole-4-carboxaldehyde, 2-quinolinecarboxaldehyde, 3-quinolinecarboxaldehyde, 4-quinolinecarboxaldehyde, 4-isoquinolinecarboxaldehyde, isoquinoline-5-carboxaldehyde, 2-thiazol-2-yl-benzaldehyde, 6-(2-thienyl)-2-pyridinecarboxaldehyde, 6-(3-thienyl)pyridine-2-carboxaldehyde, 5-phenylisoxazole-3-carboxaldehyde, 3-phenylisoxazole-5-carboxaldehyde, 8-hydroxy-2-quinolinecarboxaldehyde, 2-amino-3-formylchromone, 1-phenyl-1H-pyrazole-4-carboxaldehyde, 3-phenyl-1H-pyrazole-4-carboxaldehyde, 4-(1H-imidazol-1-yl)benzaldehyde, 3-methylbenzo[b]thiophene-2-carboxaldehyde, 5-(5-methylthiophen-2-

yl)thiophene-2-carbaldehyde, 3-(prop-2-yn-1-yloxy)benzaldehyde, 2-formylcinnamic acid, 4-formylcinnamic acid, 6-hydroxychromene-3-carboxaldehyde, 2-(4-bromo-2-formylphenoxy)propanoic acid, (2-bromo-4-formyl-6-methoxyphenoxy)acetic acid, (2-chloro-4-formyl-6-methoxy-phenoxy)-acetic acid, (5-chloro-4-formyl-2-methoxy-phenoxy)-acetic acid, 1-methylindole-3-carboxaldehyde, 1-methylindole-2-carboxaldehyde, 1-methyl-1H-indole-6-carboxaldehyde, 2-methylindole-3-carboxaldehyde, 2-methylindole-7-carboxaldehyde, 5-methylindole-3-carboxaldehyde, 5-methoxyindole-3-carboxaldehyde, 3-methyl-1H-indole-2-carboxaldehyde, 5-formyl-2-furanboronic acid MIDA, α-methyl-*trans*-cinnamaldehyde, 2-allyloxybenzaldehyde, 3-allyl-4-hydroxybenzaldehyde, 3-allylsalicylaldehyde, 4-allyloxybenzaldehyde, 4-hydroxy-3-methoxycinnamaldehyde, 2-(4-formylphenoxy)propanoic acid, vanillin acetate, (4-formyl-2-methoxyphenoxy)acetic acid, 4-(2-bromoethoxy)-3-methoxybenzaldehyde, 4-(3-fluoropropoxy)benzenecarbaldehyde, 3-bromo-2-isopropoxy-5-formylphenylboronic acid, 2,4,5-trimethylbenzaldehyde, 4-iso-propylbenzaldehyde, 2-iso-propylbenzaldehyde, 3-iso-propylbenzaldehyde, 3-phenylbutyraldehyde, 4-propylbenzaldehyde, mesitaldehyde, 2,3-dimethyl-4-methoxybenzaldehyde, 2,5-dimethyl-4-methoxybenzaldehyde, 3-(4-methoxyphenyl)propionaldehyde, 3-benzyloxypropionaldehyde, 4-propoxybenzaldehyde, 2,4-dimethoxy-3-methylbenzaldehyde, 2,4-dimethoxy-6-methylbenzaldehyde, 2-ethoxy-3-methoxybenzaldehyde, 3-ethoxy-4-methoxybenzaldehyde, 4-ethoxy-3-methoxybenzaldehyde, 2,3,4-trimethoxybenzaldehyde, 2,4,5-trimethoxybenzaldehyde, 2,4,6-trimethoxybenzaldehyde, 3,4,5-trimethoxybenzaldehyde, 2-ethoxy-3-formyl-5-methylphenylboronic acid, 3-formyl-4-isopropoxyphenylboronic acid, 3-formyl-5-isopropoxyphenylboronic acid, 3-formyl-5-propoxyphenylboroni, 3-[(dimethylamino)methyl]-4-hydroxybenzaldehyde, N-methyl-N-(2-hydroxyethyl)-4-aminobenzaldehyde, N-boc-pyrrole-2-carboxaldehyde, (1R)-(−)-myrtenal, *(S)*-(−)-perillaldehyde, alpha-cyclociral, citral, *trans,trans*-2,4-decadienal, Boc-L-prolinal, Boc-D-prolinal, citronellal, *cis*-7-decenal, *cis*-4-decenal, decanal, 7-hydroxycitronellal, 6-chloro[1,3]dioxolo[4,5-g]quinoline-7-carboxaldehyde, 5-(4-chloro-2-nitrophenyl)furfural, 5-(2,4-dichlorophenyl)furfural, 5-(2,5-dichlorophenyl)furfural, 5-(3,4-dichlorophenyl)furfural, 1-bromo-2-naphthaldehyde, 5-(4-bromophenyl)furfural, 5-(2-chlorophenyl)furfural, 5-(3-chlorophenyl)furfural, 5-(4-chlorophenyl)furfural, 4-chloro-3-formyl-6-methylcoumarin, 6-chloro-3-formyl-7-methylchromone, 4-fluoro-1-naphthaldehyde, 1-nitro-2-naphthaldehyde, 5-(2-nitrophenyl)furfural, 3-(4-bromophenyl)-5-methylisoxazole-4-carboxaldehyde, 2-chloro-6-methylquinoline-3-carboxaldehyde, 2-chloro-8-methylquinoline-3-carboxaldehyde, 3-(4-chlorophenyl)-5-methylisoxazole-4-carboxaldehyde, 3-(4-fluorophenyl)-5-methylisoxazole-4-carboxaldehyde, 5-(4-formylphenyl)pyrimidine, 1-naphthaldehyde, 2-naphthaldehyde, 4-(2-thienyl)benzaldehyde, 4-phenylthiophene-2-carboxaldehyde, 5-phenylthiophene-2-carboxaldehyde, 1-hydroxy-2-naphthaldehyde, 2-hydroxy-1-naphthaldehyde, 4-hydroxy-1-naphthaldehyde, 5-phenyl-2-furaldehyde, 3-formyl-6-methylchromone, 6-methoxychromone-3-carboxaldehyde, 1-(4-bromophenyl)-5-methyl-1H-pyrazole-4-carboxaldehyde, 1-(4-fluorophenyl)-5-methyl-1H-pyrazole-4-carboxaldehyde, 5-chloro-3-methyl-1-phenyl-4-pyrazolecarboxaldehyde, 1-acetyl-3-indolecarboxaldehyde, (3-formyl-1-indolyl)acetic acid, 5-(4-methoxyphenyl)isoxazole-3-carboxaldehyde, 1-(phenylsulfonyl)-2-pyrrolecarboxaldehyde, 5-(4-methylphenyl)isoxazole-3-carboxaldehyde, 5-methyl-3-phenylisoxazole-4-carboxaldehyde, 5-methyl-1-phenyl-1H-pyrazole-4-

carboxaldehyde, 3-methyl-1-phenyl-1H-pyrazole-4-carboxaldehyde, 4-(4-hydroxybut-1-yn-1-yl)benzaldehyde, methyl 4-formylcinnamate, (2-chloro-6-ethoxy-4-formylphenoxy)acetic acid, 5-methoxy-4-methylindole-3-carboxaldehyde, 4-[(2-cyanoethyl)methylamino]benzaldehyde, 6-methoxyindane-5-carbaldehyde, *trans*-3,5-dimethoxy-4-hydroxycinnamaldehyde, methyl 2-formyl-3,5-dimethoxybenzoate, 4-[bis-(2-chloroethyl)amino]benzaldehyde, 4-(1-pyrrolidino)benzaldehyde, 4-(dimethylamino)cinnamaldehyde, 4-(4-morpholinyl)benzaldehyde, 3-[(benzyloxycarbonyl)amino]propionaldehyde, 4-butoxy-3-nitrobenzaldehyde, n-(3-formyl-2-pyridinyl)-2,2-dimethylpropanamide, 2,3,5,6-tetramethylbenzaldehyde, 4-butylbenzaldehyde, 4-isobutylbenzaldehyde, 4-*tert*-butylbenzaldehyde, 2-(*tert*-butylthio)benzaldehyde, (r,s)-3-benzyloxy-2-methylpropionaldehyde, 3-*tert*-butyl-2-hydroxybenzaldehyde, 4-(*tert*-butyloxy)benzaldehyde, 4-butoxybenzaldehyde, 2,3-diethoxybenzaldehyde, 2,4-diethoxybenzaldehyde, 2,5-diethoxybenzaldehyde, 3,4-diethoxybenzaldehyde, 5-*tert*-butyl-2-hydroxybenzaldehyde, 3-butoxy-5-formylphenylboronic acid, 3-formyl-2-isopropoxy-5-methylphenylboronic acid, 3-formyl-5-methyl-2-propoxyphenylboronic acid, 4-diethylaminobenzaldehyde, 4-(diethylamino)salicylaldehyde, 4-[N,N-bis(2-hydroxyethyl)amino]benzaldehyde, hexylthiophene-2-carboxaldehyde, 2,6,6-trimethyl-1-cyclohexene-1-acetaldehyde, 1-boc-piperidine-4-carboxaldehyde, N-boc-cycloleucinal, *(R)*-(+)-3-boc-2,2-dimethyloxazolidine-4-carboxaldehyde, undecanal, 5-[2-chloro-5-(trifluoromethyl)phenyl]furfural, 5-[2-(trifluoromethyl)phenyl]furfural, 2-chloro-5-phenylpyridine-3-carboxaldehyde, 6-(4-chlorophenyl)-2-pyridinecarboxaldehyde, 6-(4-fluorophenyl)-2-pyridinecarboxaldehyde, 5-[3-(trifluoromethyl)phenyl]furfural, 6-(3-nitrophenyl)-2-pyridinecarboxaldehyde, 5-(3-chloro-4-methoxyphenyl)furfural, 4-(2-pyridyl)benzaldehyde, 4-(4-formylphenyl)pyridine, 4-(pyridin-3-yl)benzaldehyde, *p*-formylbenzoic acid N-hydroxysuccinimide ester, 2-methoxy-1-naphthaldehyde, 4-methoxy-1-naphthaldehyde, 2-methyl-1-naphthaldehyde, 6-methoxy-2-naphthaldehyde, 6-ethyl-3-formylchromone, 2-formylphenylboronic acid MIDA ester, 3-formylphenylboronic acid MIDA ester, 4-formylphenylboronic acid MIDA ester, 1-(4-methoxyphenyl)-5-methyl-1h-pyrazole-4-carboxaldehyde, 4-antipyrinecarboxaldehyde, 4-(3-hydroxy-3-methylbut-1-yn-1-yl)benzaldehyde, 4-acetoxy-3-methoxycinnamaldehyde, 6-isopropylindole-3-carboxaldehyde, 6-isopropylindole-3-carboxaldehyde, 4-(1-piperidinyl)benzaldehyde, 2-(boc-amino)benzaldehyde, 4-(boc-amino)benzaldehyde, pentamethylbenzaldehyde, 3-(diethoxymethyl)benzaldehyde, 4-(diethoxymethyl)benzaldehyde, 2-butoxy-3-formyl-5-methylphenylboronic acid, 4-[3-(dimethylamino)propoxy]benzaldehyde, N-boc-4-piperidineacetaldehyde, dodecyl, aldehyde name, 4′-chloro-3′-fluoro[1,1′-biphenyl]-2-carbaldehyde, 3′,4′-dichloro[1,1′-biphenyl]-2-carbaldehyde, 3-(3,4-dichlorophenoxy)benzaldehyde, 3-(3,5-dichlorophenoxy)benzaldehyde, 4-(2,4-dichlorophenoxy)benzaldehyde, 4-(6-formylpyridin-2-yl)benzonitrile, 3-(6-formylpyridin-2-yl)benzonitrile, dibenzofuran-4-carboxaldehyde, 4-(4-bromophenoxy)benzaldehyde, 2′-chloro-biphenyl-2-carbaldehyde, 3-(4-chlorophenyl)benzaldehyde, 4′-chloro-biphenyl-4-carbaldehyde, 2-(4-chlorophenoxy)benzaldehyde, 3-(4-chlorophenoxy)benzaldehyde, 4-(3-chlorophenoxy)benzaldehyde, 4-(4-chlorophenoxy)benzaldehyde, 2-(4-fluorophenyl)benzaldehyde, 2-fluorobiphenyl-4-carboxaldehyde, 4-(4-fluorophenoxy)benzaldehyde, 4-fluoro-3-phenoxybenzaldehyde, 4-(4-nitrophenoxy)benzaldehyde, 1H-benzo[g]indole-3-carboxaldehyde, 5-acenaphthenecarboxaldehyde, biphenyl-2-carboxaldehyde, biphenyl-3-carboxaldehyde, biphenyl-4-

carboxaldehyde, 3-phenoxybenzaldehyde, 4-phenoxybenzaldehyde, 2-(4-formylphenyl)-6-(hydroxymethyl)pyridine, 6-(4-methoxyphenyl)-2-pyridinecarboxaldehyde, 6-[4-(methylsulfonyl)phenyl]-2-pyridinecarboxaldehyde, 2,3-dimethoxy-1-naphthaldehyde, 3,8-dimethoxy-2-naphthaldehyde, 4,7-dimethoxy-1-naphthaldehyde, 4-dimethylamino-1-naphthaldehyde, 1-boc-7-azaindole-3-carboxaldehyde, 3-(1-Methyl-1H-indol-3-yl)butyraldehyde, 2,6-difluoro-4-formylphenylboronic acid, 2-fluoro-5-formylphenylboronic acid pinacol ester, 2-(1,3,3-trimethylindolin-2-ylidene)acetaldehyde, 2,3,6,7-tetrahydro-8-hydroxy-1h,5h-benzo[ij]quinolizine-9-carboxaldehyde, 2-methyl-N-ethyl-n-(2-cyanoethyl)-4-aminobenzaldehyde, 2-cyclohexylbenzaldehyde, N-benzylpiperidine-4-carboxaldehyde, *trans*-4-(diethylamino)cinnamaldehyde, 2-methyl-3-phenylpropanal, 3-(4-*tert*-butylphenyl)propanal, 2-(hexyloxy)benzaldehyde, 4-(hexyloxy)benzaldehyde, 4-(4-hydroxy-4-methylpentyl)-3-cyclohexene-1-carboxaldehyde, tridecanal, 3-formylphenyl 2,4-dichlorobenzoate, 3-formylphenyl 2-chlorobenzoate, 3-formylphenyl 4-chlorobenzoate, 2′-trifluoromethyl-biphenyl-4-carbaldehyde, 2-[4-(trifluoromethyl)phenyl]benzaldehyde, 3′-(trifluoromethyl)-1,1′-biphenyl-2-carboxaldehyde, 3-[3-(trifluoromethyl)phenoxy]benzaldehyde, 4-(4-formylphenoxy)benzonitrile, fluorene-2-carboxaldehyde, 4-(4-bromobenzyloxy)benzaldehyde, methyl 3-(6-formyl-2-pyridinyl)benzoate, methyl 4-(6-formylpyridin-2-yl)benzoate, 3-(4-methylphenyl)benzaldehyde, 4-(4-methylphenyl)benzaldehyde, diphenylacetaldehyde, 2′-methoxy-biphenyl-2-carbaldehyde, 2-benzyloxybenzaldehyde, 3-benzyloxybenzaldehyde, 4-benzyloxybenzaldehyde, 3-(4-methylphenoxy)benzaldehyde, 4-(4-methylphenoxy)benzaldehyde, 2-(3-methoxyphenoxy)benzaldehyde, 2-(4-methoxyphenoxy)benzaldehyde, 3-(4-methoxyphenoxy)benzaldehyde, 4-(4-methoxyphenoxy)benzaldehyde, 4-(benzyloxy)-2-hydroxybenzaldehyde, 2-[(phenylsulfonyl)methyl]benzaldehyde, N-boc-indoline-7-carboxaldehyde, α-amylcinnamaldehyde, N-Boc-phenylalaninal, 2-(4-*tert*-butylbenzyl)propionaldehyde, 4-(heptyloxy)benzaldehyde, 3-isobutylaziridine-2-carboxaldehyde, 4-[3,5-bis(trifluoromethyl)phenyl]benzaldehyde, 9-anthracenecarboxaldehyde, 10-chloro-9-anthraldehyde, 9-phenanthrenecarboxaldehyde, phenanthrene-4-carbaldehyde, 3-(2-furoyl)quinoline-2-carboxaldehyde, *trans*-4-stilbenecarboxaldehyde, 4′-bromostilbene-4-carboxaldehyde, 2-methyl-9-acridinecarboxaldehyde, 2-phenylindole-3-carboxaldehyde, β-phenylcinnamaldehyde, methyl 4-(3-formylphenyl)benzoate, methyl 4-(4-formylphenyl)benzoate, 9-ethyl-3-carbazolecarboxaldehyde, 2-benzyloxy-3-methoxybenzaldehyde, 3-benzyloxy-4-methoxybenzaldehyde, 4-benzyloxy-3-methoxybenzaldehyde, [3a*R*(3aα,4α,5β,6aα)]-(−)-5-(benzoyloxy)hexahydro-2-oxo-2H-cyclopenta[b]furan-4-carboxaldehyde, 4-[bis[2-(acetyloxy)ethyl]amino]benzaldehyde, α-hexylcinnamaldehyde, 4′-formylbenzo-15-crown-5, 3,5-di-*tert*-butylbenzaldehyde, 3,5-di-*tert*-butyl-2-hydroxybenzaldehyde, 3,5-di-*tert*-butyl-4-hydroxybenzaldehyde, 4-octylbenzaldehyde, 4-(dibutylamino)benzaldehyde, farnesal, oncrasin-1, 1-benzylindole-3-carboxaldehyde, 5-benzyloxyindole-3-carboxaldehyde, 1,3-diphenyl-1h-pyrazole-4-carboxaldehyde, 10-methylanthracene-9-carboxaldehyde, 4-benzyloxy-3,5-dimethylbenzaldehyde, 1-boc-4-(4-formylphenyl)piperazine, 1-boc-4-(2-formylphenyl)piperazine, 3,5-di-*tert*-butyl-2-methoxybenzaldehyde, *cis*-11-hexadecenal, 1-pyrenecarboxaldehyde, 3-(4-*tert*-butylphenoxy)benzaldehyde, 4-(decyloxy)benzaldehyde, 3-(1-methylindol-3-yl)-3-phenylpropionaldehyde, 4-(diphenylamino)benzaldehyde, 2-(diphenylphosphino)benzaldehyde, 3-

(3′,5′-di-*tert*-butyl-4′-hydroxy)phenyl-2,2-dimethylpropanal, 3,5-dibenzyloxybenzaldehyde, 3,4-dibenzyloxybenzaldehyde.

S2. Full list of enzymes and the best inhibitors

Table S1. The full list of enzymes with preferred P1′ substituents that were the most favored, had the best interaction with the catalytic metal ion via the phosphinate group, and were the most specific. The stereochemistry of the ligands and the calculated Gibbs free energy of binding in the active center are included. Known inhibitors are given as the references, with inhibition potency (K_i or IC50) and calculated ΔG values.

Enzyme	Inhibitor	P1′ substituent	Configuration	ΔG [kJ/mol]
M01.001 *Homo sapiens* 4FYT	The best found	4-Hydroxy-3-methoxyphenyl	*S,S*	-53.82
	Metal-interacting			
	The most specific	5-Amino-thiophen-2-yl	*R,S*	-34.19
	Known, K_i = 0.69 nM	R = 4-(Aminomethyl)phenyl [1]		-64.11
M01.001 *Sus scrofa* 4FKE	The best found	4-Boronophenyl MIDA ester	*R,R*	-39.09
	Metal-interacting			
	The most specific			
	Known, IC_{50} = 8.10 μM	Bestatin [2]		-45.38
M01.003 *Homo sapiens* 4KX7	The best found	3-Isobutylaziridin-2-yl	*R,S*	-38.80
	Metal-interacting			
	The most specific	1-Methyl-1H-imidazol-5-yl	*R,S*	-26.34
	Known, K_i = 75 μM	Bestatin [3]		-7.54
M01.004 *Homo sapiens* 4MKT	The best found	2-Borono-5-methoxyphenyl	*R,S*	-57.39
	Metal-interacting	none		
	The most specific	2-Butyl-5-chloro-1H-imidazol-4-yl	*R,S*	-40.94
	Known, K_i = 200 nM	Bestatin [4]		-53.73
M01.005 *Escherichia coli* 3B34	The best found	3,4-Dihydroxyphenyl	*R,E*	-49.91
	Metal-interacting			
	The most specific	none		
	Known, K_i = 1.5 nM	Ala-P[(O)(OH)CH_2]-Phe-Phe [5]		-50.47
M01.011 *Homo sapiens* 5MJ6	The best found	3-(Furan-2-carbonyl)quinolin-2-yl	*R,S*	-117.58
	Metal-interacting			
	The most specific	none		
	Known, K_i = 1.7 nM	IVDE77 [6]		-53.58
M01.021 *Thermoplasma acidophilum* 1Z5H	The best found	4-(6-(Hydroxymethyl)pyridin-2-yl)phenyl	*R,S*	-37.43
	Metal-interacting	6-Ethyl-4-oxo-4*H*-chromen-3-yl	*R,S*	-37.15
	The most specific			-37.15
	Unknown	Bestatin		-6.42
M01.024	The best found	2-Benzyloxyphenyl	*R,S*	-76.49

Homo sapiens 5AB0	Metal-interacting	Phenanthren-4-yl	*R*,*Z*	-36.15
	The most specific	Diphenylmethyl	*R*,*Z*	-52.41
	Known, IC_{50} = 237 nM	DABA-Trp derivative [7]		-54.74
M01.029 *Plasmodium falciparum* 3EBH	The best found	3-Hydroxy-4-methoxyphenyl	*R*,*S*	-63.40
	Metal-interacting			
	The most specific	4-(2-Pyridyl)phenyl	*R*,*E*	-63.35-
	Known, IC_{50} = 6 nM	BDM14471 [8]		-37.38
M01.031 *Colwellia psychrerythraea* 3CIA	The best found / Metal-interacting / The most specific	2-(*tert*-Butylthio)phenyl	*R*,*R*	-76.28
	Unknown value	Bestatin		-66.93
M01.034 *Saccharomyces cerevisiae* 2XQ0	The best found / Metal-interacting	3,5-Di-*tert*-butylphenyl	*S*,*R*	-53.62
	The most specific	3-phenyl-1H-pyrazol-4-yl	*R*,*R*	-44.73
	Unknown	Bestatin		-47.06
M01.un *Neisseria meningitides* 2GTQ	The best found / Metal-interacting	Quinolin-4-yl	*R*,*E*	-54.98
	The most specific	Biphenyl-2-yl	*R*,*E*	-45.78
	Known, K_i = 54 nM	R = 4-Hydroxyphenyl [9]		-26.61
M17.001 *Bos taurus* 1LAM	The best found	3-(4-Chlorophenyl)phenyl		-90.35
	Metal-interacting / The most specific	4,6-Dichloro-2H-chromen-3-yl		-82.09
	Known, K_i = 22 nM	L-leucinthiol [10]		-40.78
M17.002 *Solanum lycopersicum* 4KSI	The best found	4-Borono-3-fluorophenyl	*R*,*R*	
	Metal-interacting	3-Boronophenyl	*R*,*S*	
	The most specific	3-Methylbenzo[b]thiophen-2-yl	*S*,*S*	
	Known, Activity = 0% for 0.1 mM inhibitor	Bestatin		-39.99
M17.003 *Escherichia coli* 1GYT	The best found / Metal-interacting	N-phenylpiperidin-4-yl	*S*,*E*	-86.01
	The most specific	2,2′-Bithiophen-5-yl	*R*,*S*	-75.42
	Unknown	Bestatin		-40.66
M17.016 *Helicobacter pylori* 4ZLA	The best found / Metal-interacting	3-Borono-6-ethoxyphenyl	*S*,*Z*	-81.94
	The most specific	None		
	Known, IC_{50} = 49 nM	Bestatin [11]		-26.60
M17.A05 *Caenorhabditis elegans* 2HC9	The best found	4-Borono-2-fluorophenyl	*R*,*R*	-78.00
	Metal-interacting	3-Borono-5-Bromo-4-methoxyphenyl	*R*,*Z*	-68.29
	The most specific	Benzofuran-2-yl	*R*,*R*	-66.70
	Unknown	Bestatin		-35.60
M17.uns	The best found	6-(3-Cyanophenyl)pyridin-2-yl	*S*,*Z*	-92.97

Pseudomonas putida 3H8G	Metal-interacting			
	The most specific			
	Unknown	Bestatin [12]		-37.17
M17.uns *Xanthomonas oryzae* 3JRU	The best found	3-Brorono-5-methylphenyl	*S,S*	-81.84
	Metal-interacting	3-Borono-2-methoxy-5-methylphenyl	*S,S*	-76.81
	The most specific	3-(4-Fluorophenyl)propyl	*R,S*	-77.93
	Unknown	Bestatin		-46.58
M18.002 *Bos Taurus* 3VAT	The best found	4-(4-cyanophenoxy)phenyl	*R,R*	-78.72
	Metal-interacting	1-(4-fluorophenyl)-5-methyl-1H-pyrazol-4-yl	*R,S*	-74.22
	The most specific	4-(4-cyanophenoxy)phenyl	*R,R*	-78.72
	Known,	Phenantroline [13]		-28.47
M18.002 *Homo sapiens* 4DYO	The best found	3,4-Dibenzyloxyphenyl	*R,S*	-88.05
	The most specific	None		
	Metal-interacting	5-(Ethoxymethyl)furan-2-yl	*S,S*	-46.49
	Known, C = 50 μg/ml – 50%	Phenantroline [13]		-16.74
M18.002 *Pseudomonas aeruginosa* 4NJR	The best found<br>Metal-interacting	Imidazo-2-yl	*S,E*	-67.88
	The most specific	Ethyl	*S,R*	-37.17
	Known	Phenantroline [13]		-16.83
M18.003 *Plasmodium falciparum* 4EME	The best found	3,5-Dihydroxyphenyl	*R,S*	-80.32
	Metal-interacting<br>The most specific	3-Cyano-4-fluorophenyl	*S,S*	-60.49
	Known, K_i = 0.34 μM	(*R*)-Glu α-phosphonic amino acid derivative [14]		-29.94
M18.004 *Clostridium acetobutylicum* 2GLJ	The best found	Acenaphthen-5-yl	*R,R*	-117.11
	Metal-interacting<br>The most specific	8-Hydroxyquinolin-yl	*R,E*	-111.17
	Unknown	Bestatin		-88.90
M18.004 *Thermotoga maritima* 2GLF	The best found<br>Metal-interacting<br>The most specific	3,4-Dichlorophenyl	*R,R*	-63.23
	Unknown	Bestatin		-31.90
M24.001 *Escherichia coli* 2GGC	The best found<br>Metal-interacting	3-Isobutylaziridin-2-yl	*S,R*	-65.96
	The most specific	4-(1H-Imidazol-1-yl)phenyl	*R,R*	-41.39
	Known, IC_{50} = 67 nM	Inhibitor 2 [15]		-28.38
M24.001 *Mycobacterium tuberculosis*	The best found	3-Borono-6-ethoxyphenyl	*R,R*	-50.98
	Metal-interacting	None		
	The most specific	3-Borono-4-methoxy-5-Bromophenyl	*R,E*	-28.25

1YJ3	Unknown value	Inhibitor 2 [15]		-46.56
M24.001 *Rickettsia prowazekii* 3MX6	The best found	Isoquinolin-5-yl	*R,E*	-77.66
	Metal-interacting	Dimethylpropyl	*S,R*	-75.66
	The most specific	Isoquinolin-5-yl	*R,E*	-77.66
	Known, IC_{50} = 0.6 μM	Furoic acid derivative (6) [16]		-42.62
M24.002 *Homo sapiens* 1B6A	The best found	4-Borono-2,3-difluorophenyl	*R,S*	-84.15
	The most specific	3-(3-Chlorophenyl)propyl	*S,S*	-55.00
	Metal-interacting	Phenylethyl	*S,S*	-59.33
	Known, K_i = 26 nM	Beloranib [17]		-28.39
M24.017 *Homo sapiens* 2GZ5	The best found	4-Boronophenyl	*R,E*	-85.18
	The most specific	4-Borono-3-fluorophenyl	*R,R*	-51.19
	Metal-interacting	4-(6-(hydroxymethyl)pyridinyl)phenyl	*S,R*	-77.96
	Known, K_i = 10 nM	Barbiturate-based inhibitor (10)[18]		-53.46
M24.035 *Pyrococcus furiosus* 1XGS	The best found			
	Metal-interacting	2-Bromo-3-pyridinecarboxaldehyde	*R,S*	-66.07
	The most specific			
	Known, K_i = 2 μM	Bestatin-like inhibitor (A311263) [19]		-32.44
M24.036 *Staphylococcus aureus* 1QXY	The best found	Benzo[1,3]dioxol-5-yl	*R,R*	-93.88
	Metal-interacting			
	The most specific	2-Methylindol-7-yl	*R,S*	-59.33
	Known, IC_{50} = 7 μM	3-Amino-1-(cyclopropylamino)heptan-2-one [20]		-34.55
M24A.uns *Encephalitozoon cuniculi* 3FM3	The best found	2-Methyl-3-phenylpropyl	*S,S,R*	-106.79
	Metal-interacting	1-(Benzyloxy)ethyl	*S,R,S*	-103.11
	The most specific			
	Known, IC_{50} = 95.0 nM	TNP-470 [21]		-61.73
M24.004 *Escherichia coli* 1WL9	The best found	bicyclo[2.2.1]heptan-2-yl	*S,S*	-80.36
	Metal-interacting	6-Methoxyindan-5-yl	*S,Z*	-57.65
	The most specific	(2-(Benzyloxycarbonyl)amino)ethyl	*S,R*	-57.93
	Known, K_i = 14 μM	Apstatin [22]		-44.43
M24.009 *Homo sapiens* 3CTZ	The best found	4-Borono-2-fluorophenyl	*R,R*	-51.91
	The most specific	2-Boronophenyl	*S,S*	-41.61
	Metal-interacting	None		
	Known, K_i = 260 nM	GW796406[1] [23]		-42.11
M24.026 *Homo sapiens* 5X49	The best found	3-Phenylaziridin-2-yl	*R,S*	-102.22
	The most specific	2-Borono-6-fluorophenyl	*R,R*	-68.93
	Metal-interacting	3-Borono-5-isopropoxyphenyl	*R,R*	-83.35
	Known, K_i = 640 nM	Apstatin [22]		-103.24
M24.031 *Thermotoga maritima*	The best found			
	Metal-interacting	4-Bromophenyl	*R,E*	-67.12
	The most specific			

2ZSG	Unknown	Bestatin		-37.08
M24.038	The best found	4-(Pyridin-3-yl)phenyl	*S,R*	-83.36
Plasmodium falciparum	Metal-interacting	1,3-Diphenyl-1H-pyrazol-4-yl	*S,E*	-67.21
	The most specific			
5JR6	Known, K_i = 20.2 μM	Apstatin [24]		-47.01
M24.A11	The best found	2-(Carboxymethoxy)-5-chlorophenyl	*S,S*	-91.54
Xanthomonas	Metal-interacting			
campestris	The most specific	5-(Ethoxycarbonyl)-1H-pyrrol-3-yl	*R,R*	-60.77
5CDE	Unknown	Bestatin		-18.85
M28.003	The best found	1H-Indazol-6-yl	*R,S*	-72.68
Streptomyces	Metal-interacting			
griseus	The most specific	2-Hydroxy-5-methoxyphenyl	*R,R*	-56.82
1CP7	Unknown	Phenantroline [25]		-33.12
M28.uns	The best found	3,4-Dihydroxyphenyl	*R,S*	-74.32
Clostridium	Metal-interacting			
acetobutylicum	The most specific	2,4-Dichloro-6-hydroxyphenyl	*R,R*	-73.68
3K9T	Unknown	Phenantroline		-12.89
M28.uns	The best found	2,3,4-Trihydroxyphenyl	*S,Z*	-63.56
Parabacteroides	Metal-interacting	None		
distasonis	The most specific	6-Hydroxychromen-3-yl	*S,R*	-37.56
3TC8	Unknown value	Phenantroline		-34.67
M28.002	The best found	3-Methylpyridin-2-yl	*R,R*	-98.84
Vibrio	Metal-interacting	2-Ethylpropyl	*S,S*	-74.28
proteolyticus	The most specific	6-Methylpyridin-2-yl	*S,R*	-92.41
1RTQ	Known, K_i = 18 nM	Bestatin [26]		-71.01
M28.020	The best found	6-Methyl-4-oxo-4H-chromen-3-yl	*S,S*	-54.56
Aneurinibacillus	Metal-interacting			
sp. AM-1	The most specific			
2EK8	Unknown	Bestatin		-59.12
M29.001	The best found	3-Borono-5-isopropoxyphenyl	*S,Z*	-64.62
Thermus	Metal-interacting	2,3-Dihydrobenzofuran-5-yl	*R,E*	-59.81
thermophiles	The most specific			
2AYI	Unknown	Bestatin		-40.69
M29.005	The best found	5-Chlorofuran-2yl	*R,R*	-72.55
Staphylococcus	Metal-interacting			
aureus	The most specific	2-Bromophenyl	*R,R*	-55.22
1ZJC	Unknown value	Bestatin		-44.63
M42.001	The best found	4-[N,N-Bis(2-hydroxyethyl)amino]phenyl	*S,Z*	-50.59
Bacillus subtilis	Metal-interacting	None		
1VHE	The most specific	None		

	Unknown value	Bestatin		-15.31
M42.001 *Streptococcus pneumoniae* 3KL9	The best found	2-Chloro-5-phenylpyridin-3-yl	*R,Z*	-67.27
	Metal-interacting			
	The most specific	4-(Difluoromethoxy)-3-hydroxyphenyl	*R,S*	-56.17
	Known, K_i = 292 nM	Bestatin[5] [27]		-41.97
M42.003 *Pyrococcus horikoshii* 2CF4	The best found	3,4-Dibenzyloxyphenyl	*S,S*	-71.91
	Metal-interacting			
	The most specific	4-(Dimethylamino)phenyl	*R,E*	-40.93
	Unknown value	Bestatin		-35.03
M42.004 *Pyrococcus horikoshii* 1Y0Y	The best found	2,3-Dimethoxynaphtha-1-yl	*R,Z*	-58.55
	Metal-interacting	4-(4-Methylphenoxy)phenyl	*R,Z*	-50.41
	The most specific			
	Unknown value	Bestatin		-27.75
M42.009 *Pyrococcus horikoshii* 2VPU	The best found	Indol-7-yl	*S,R*	-57.37
	Metal-interacting	3-Iodo-4-hydroxy-5-methoxyphenyl	*S,R*	-51.41
	The most specific			
	Unknown value	Bestatin		-42.42
M55.001 *Bacillus subtilis* 1HI9	The best found	Indol-4-yl	*S,R*	-86.11
	Metal-interacting			
	The most specific	Biphen-3-yl	*S,Z*	-68.89
	Unknown value	Bestatin		-55.27

S3. ADMET properties of the best selected inhibitors from the Table S1.

Table S2. Selected ADMET properties for the best inhibitors: MW – molecular weight, H-acc – H-bond acceptor number, H-don – H-bond donor number, LogP – logarithm of partition coefficient, GI – gastrointestinal absorption, Lipinski – if the compound does not fulfill basics of the rule of five the outside value is mentioned.

P1′ group	MW	H-acc	H-don	LogP	Water solubility	GI	Lipinski
4-Hydroxy-3-methoxyphenyl	406.39	6	2	0.5	Moderately soluble	Low	Yes
5-Amino-thiophen-2-yl	381.41	4	2	0.16	Soluble	Low	Yes
4-Boronophenyl MIDA ester	515.28	8	1	-1.61	Moderately soluble	Low	1 (MW)
3-Isobutylaziridin-2-yl	368.41	4	2	-1.13	Soluble	High	Yes
1-Methyl-1H-imidazol-5-yl	365.36	4	2	-0.64	Soluble	High	Yes
2-Borono-5-methoxyphenyl	434.21	7	3	-1.07	Soluble	Low	Yes
2-Butyl-5-chloro-1H-imidazol-4-yl	440.88	5	2	1.19	Poorly soluble	Low	Yes
3,4-Dihydroxyphenyl	390.35	6	3	0.12	Soluble	Low	Yes

3-(Furan-2-carbonyl)quinolin-2-yl	505.48	7	1	1.62	Poorly soluble	Low	1 (MW)
4-(6-(Hydroxymethyl)pyridin-2-yl)phenyl	467.47	6	2	1.02	Poorly soluble	Low	Yes
6-Ethyl-4-oxo-4H-chromen-3-yl	456.45	6	1	1.33	Poorly soluble	Low	Yes
2-Benzyloxyphenyl	466.49	5	1	2.1	Poorly soluble	High	Yes
Diphenylmethyl	448.47	4	1	2.34	Poorly soluble	High	Yes
Phenanthren-4-yl	458.47	4	1	2.67	Poorly soluble	High	Yes
3-Hydroxy-4-methoxyphenyl	406.39	6	2	0.38	Moderately soluble	Low	Yes
4-(2-Pyridyl)benzaldehyde	435.43	5	1	1.38	Poorly soluble	High	Yes
2-(*tert*-Butylthio)phenyl	448.54	4	1	2.09	Poorly soluble	Low	Yes
3,5-Di-*tert*-butylphenyl	472.58	4	1	3.28	Poorly soluble	High	Yes
3-Phenyl-1H-pyrazol-4-yl	426.43	5	2	0.77	Poorly soluble	Low	Yes
Quinolin-4-yl	409.39	5	1	1.04	Moderately soluble	High	Yes
Biphenyl-2-yl	434.44	4	1	2.27	Poorly soluble	High	Yes
3-(4-Chlorophenyl)phenyl	471.91	5	3	3.82	Poorly soluble	Low	Yes
4,6-Dichloro-2H-chromen-3-yl	484.31	6	3	3.02	Poorly soluble	Low	Yes
4-Borono-3-fluorophenyl	423.18	8	5	0.33	Moderately soluble	Low	Yes
3-Boronophenyl	405.19	7	5	0.03	Soluble	Low	Yes
3-Methylbenzo[b]thiophen-2-yl	431.48	5	3	3.2	Poorly soluble	Low	Yes
N-phenylpiperidin-4-yl	442.49	5	3	2.41	Moderately soluble	High	Yes
2,2′-Bithiophen-5-yl	449.52	5	3	3.24	Poorly soluble	Low	Yes
3-Borono-6-ethoxyphenyl	447.23	8	5	0.24	Moderately soluble	Low	Yes
4-Borono-2-fluorophenyl	423.18	8	5	0.33	Moderately soluble	Low	Yes
3-Borono-5-Bromo-4-methoxyphenyl	512.1	8	5	0.51	Moderately soluble	Low	1 (MW)
Benzofuran-2-yl	401.39	6	3	2.33	Moderately soluble	High	Yes
6-(3-Cyanophenyl)pyridin-2-yl	460.44	7	2	1.63	Poorly soluble	Low	Yes
3-Brorono-5-methylphenyl	419.22	7	5	0.32	Moderately soluble	Low	Yes
3-Borono-2-methoxy-5-methylphenyl	449.24	8	5	0.28	Moderately soluble	Low	Yes
3-(4-Fluorophenyl)propyl	407.42	6	3	2.98	Poorly soluble	High	Yes
4-(4-cyanophenoxy)phenyl	478.48	7	3	3.03	Poorly soluble	Low	Yes

1-(4-fluorophenyl)-5-methyl-1H-pyrazol-4-yl	459.45	7	3	2.52	Poorly soluble	Low	Yes
3,4-Dibenzyloxyphenyl	573.62	7	3	4.32	Insoluble	Low	1 (MW)
5-(Ethoxymethyl)furan-2-yl	409.41	7	3	1.56	Moderately soluble	High	Yes
Imidazo-2-yl	349.32	6	4	0.46	Soluble	High	Yes
Ethyl	313.33	5	3	1.34	Soluble	High	Yes
3,5-Dihydroxyphenyl	393.37	7	5	1.1	Soluble	Low	Yes
3-Cyano-4-fluorophenyl	404.37	7	3	1.84	Moderately soluble	Low	Yes
Acenaphthen-5-yl	437.47	5	3	3.18	Poorly soluble	High	Yes
8-Hydroxyquinolin-yl	428.42	7	4	1.64	Moderately soluble	Low	Yes
3,4-Dichlorophenyl	430.26	5	3	3.06	Poorly soluble	High	Yes
3-Isobutylaziridin-2-yl	368.41	6	4	0.88	Soluble	High	Yes
4-(1H-Imidazol-1-yl)phenyl	427.43	6	3	1.76	Moderately soluble	High	Yes
3-Borono-6-ethoxyphenyl	449.24	8	5	0.27	Moderately soluble	Low	Yes
3-Borono-4-methoxy-5-Bromophenyl	512.1	8	5	0.51	Moderately soluble	Low	1 (MW)
Isoquinolin-5-yl	410.4	6	3	1.95	Moderately soluble	High	Yes
Dimethylpropyl	354.4	4	1	1.4	Moderately soluble	High	Yes
4-Borono-2,3-difluorophenyl	441.17	9	5	0.63	Moderately soluble	Low	Yes
3-(3-Chlorophenyl)propyl	422.86	4	1	2.04	Poorly soluble	High	Yes
Phenylethyl	374.39	4	1	1.27	Moderately soluble	High	Yes
4-Boronophenyl	402.17	6	3	-1.06	Soluble	Low	Yes
4-Borono-3-fluorophenyl	422.17	7	3	-0.73	Moderately soluble	Low	Yes
4-(6-(hydroxymethyl)pyridinyl)phenyl	468.48	7	4	1.99	Poorly soluble	Low	Yes
2-Bromo-3-pyridinecarboxaldehyde	441.26	6	3	1.76	Moderately soluble	High	Yes
Benzo[1,3]dioxol-5-yl	425.46	7	3	1.85	Soluble	High	Yes
2-Methylindol-7-yl	413.43	4	2	1.32	Poorly soluble	High	Yes

2-Methyl-3-phenylpropyl	403.45	5	3	2.72	Moderately soluble	High	Yes
1-(Benzyloxy)ethyl	419.45	6	3	2.24	Moderately soluble	High	Yes
bicyclo[2.2.1]heptan-2-yl	379.43	5	3	2.32	Soluble	High	Yes
6-Methoxyindan-5-yl	429.45	6	3	2.54	Moderately soluble	High	Yes
(2-(Benzyloxycarbonyl)amino)ethyl	462.48	7	4	1.92	Moderately soluble	Low	Yes
4-Borono-2-fluorophenyl	423.18	8	5	0.33	Moderately soluble	Low	Yes
2-Boronophenyl	405.19	7	5	0.03	Soluble	Low	Yes
3-Phenylaziridin-2-yl	402.42	6	4	1.09	Moderately soluble	High	Yes
2-Borono-6-fluorophenyl	423.18	8	5	0.33	Moderately soluble	Low	Yes
3-Borono-5-isopropoxyphenyl	463.27	8	5	0.52	Moderately soluble	Low	Yes
4-Bromophenyl	440.27	5	3	2.73	Moderately soluble	High	Yes
4-(Pyridin-3-yl)phenyl	438.46	6	3	2.48	Poorly soluble	High	Yes
1,3-Diphenyl-1H-pyrazol-4-yl	503.53	5	4	2.81	Poorly soluble	High	1 (MW)
2-(Carboxymethoxy)-5-chlorophenyl	469.85	8	4	1.93	Moderately soluble	Low	Yes
5-(Ethoxycarbonyl)-1H-pyrrol-3-yl	422.41	7	4	1.41	Moderately soluble	Low	Yes
1H-Indazol-6-yl	401.4	6	4	1.62	Moderately soluble	Low	Yes
2-Hydroxy-5-methoxyphenyl	407.4	7	4	1.35	Moderately soluble	Low	Yes
3,4-Dihydroxyphenyl	393.37	7	5	1.11	Soluble	Low	Yes
2,4-Dichloro-6-hydroxyphenyl	446.26	6	4	2.47	Moderately soluble	Low	Yes
2,3,4-Trihydroxyphenyl	407.35	8	6	0.74	Soluble	Low	1 (NH or OH>5)
6-Hydroxychromen-3-yl	431.42	7	4	1.73	Moderately soluble	Low	Yes
3-Methylpyridin-2-yl	376.39	6	3	1.5	Moderately soluble	High	Yes
2-Ethylpropyl	355.41	5	3	2.33	Moderately soluble	High	Yes

6-Methylpyridin-2-yl	376.39	6	3	1.43	Moderately soluble	High	Yes
6-Methyl-4-oxo-4H-chromen-3-yl	443.43	7	3	2.14	Poorly soluble	Low	Yes
3-Borono-5-isopropoxyphenyl	461.25	8	5	0.49	Moderately soluble	Low	Yes
2,3-Dihydrobenzofuran-5-yl	401.39	6	3	1.93	Moderately soluble	High	Yes
5-Chlorofuran-2yl	385.78	6	3	1.75	Moderately soluble	High	Yes
2-Bromophenyl	440.27	5	3	2.58	Moderately soluble	High	Yes
4-[N,N-Bis(2-hydroxyethyl)amino]phenyl	450.42	8	6	0.52	Soluble	Low	1 (NH or OH>5)
2-Chloro-5-phenylpyridin-3-yl	394.79	6	3	1.65	Moderately soluble	High	Yes
4-(Difluoromethoxy)-3-hydroxyphenyl	443.38	9	4	2.11	Moderately soluble	Low	Yes
4-(Dimethylamino)phenyl	402.42	5	3	1.86	Moderately soluble	High	Yes
2,3-Dimethoxynaphtha-1-yl	469.47	7	3	2.77	Poorly soluble	Low	Yes
4-(4-Methylphenoxy)phenyl	465.48	6	3	3.48	Poorly soluble	Low	Yes
Indol-7-yl	400.41	5	4	1.96	Moderately soluble	High	Yes
3-Iodo-4-hydroxy-5-methoxyphenyl	533.29	7	4	2.07	Moderately soluble	Low	1 (MW)
Indol-4-yl	400.41	5	4	2.06	Moderately soluble	High	Yes
Biphen-3-yl	435.45	5	3	3.29	Poorly soluble	High	Yes

S4. Additional figures mentioned in the main text

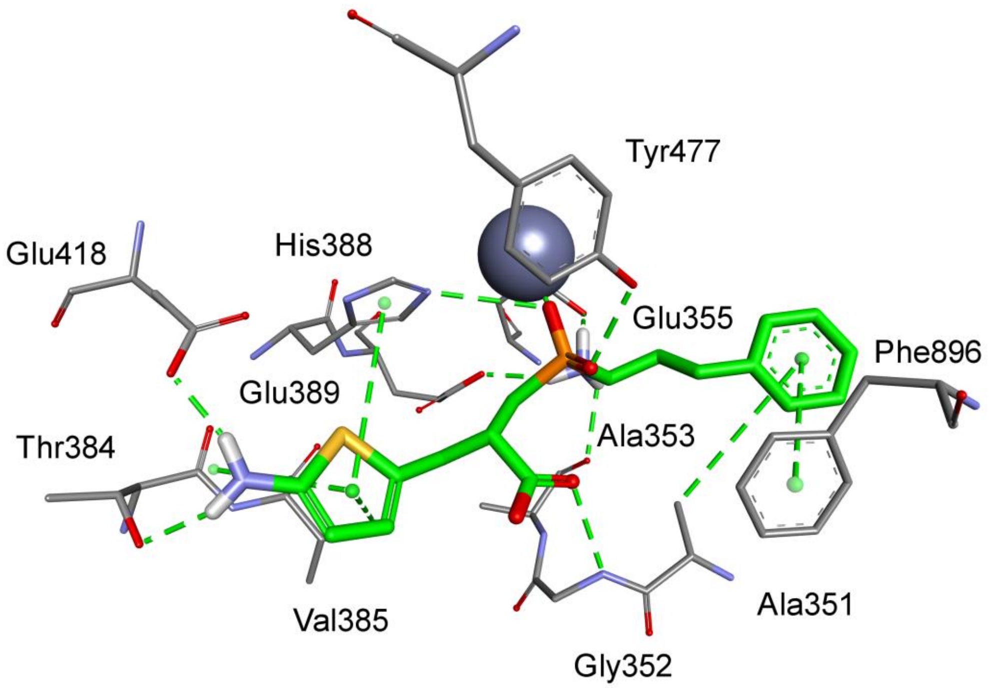

Figure S1. Modeled binding mode of a phosphinic dipeptide analog containing a 5-amino-thiophen-2-yl P1′ fragment in the active center of human aminopeptidase N (PDB: 4FYT). Inhibitor and enzyme amino acid residues are shown as sticks, while zinc ion as a grey sphere. Hydrogen bonds and non-polar interactions are shown as thin dashed lines.

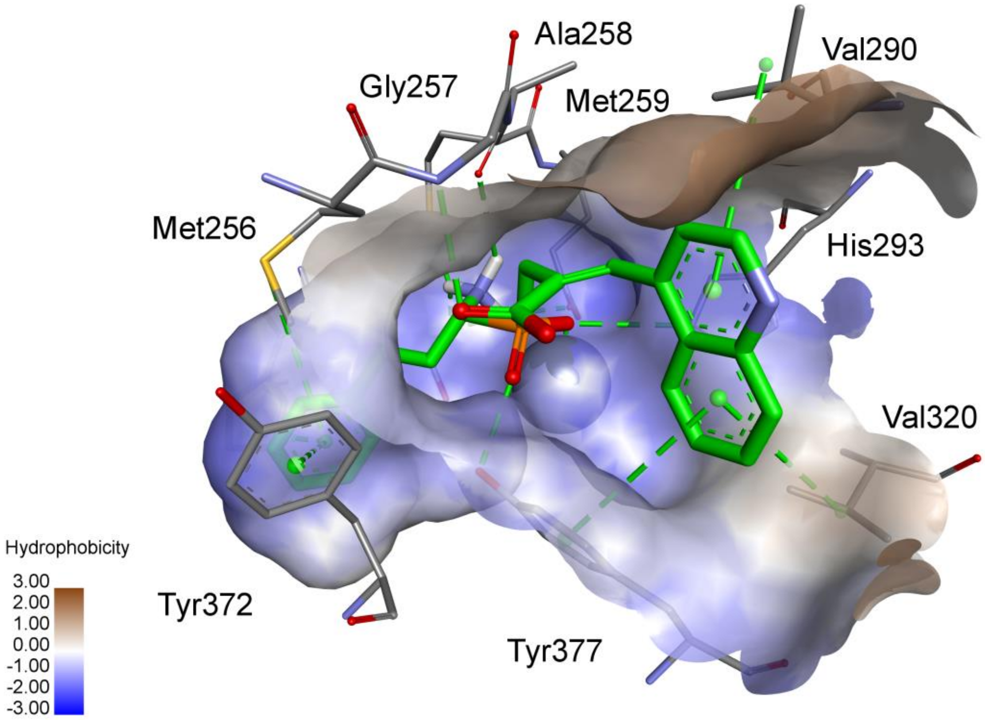

Figure S2. Modeled binding mode of a phosphinic dipeptide analog containing a quinolin-4-yl P1′ fragment in the active center of *Neisseria meningitides* aminopeptidase (PDB: 2GTQ). Inhibitor and enzyme amino acid residues are shown as sticks, while zinc ion as a grey sphere. Hydrogen bonds and non-polar interactions are shown as thin dashed lines. Surface represents the hydrophobicity of side chains the cavity amino acids.

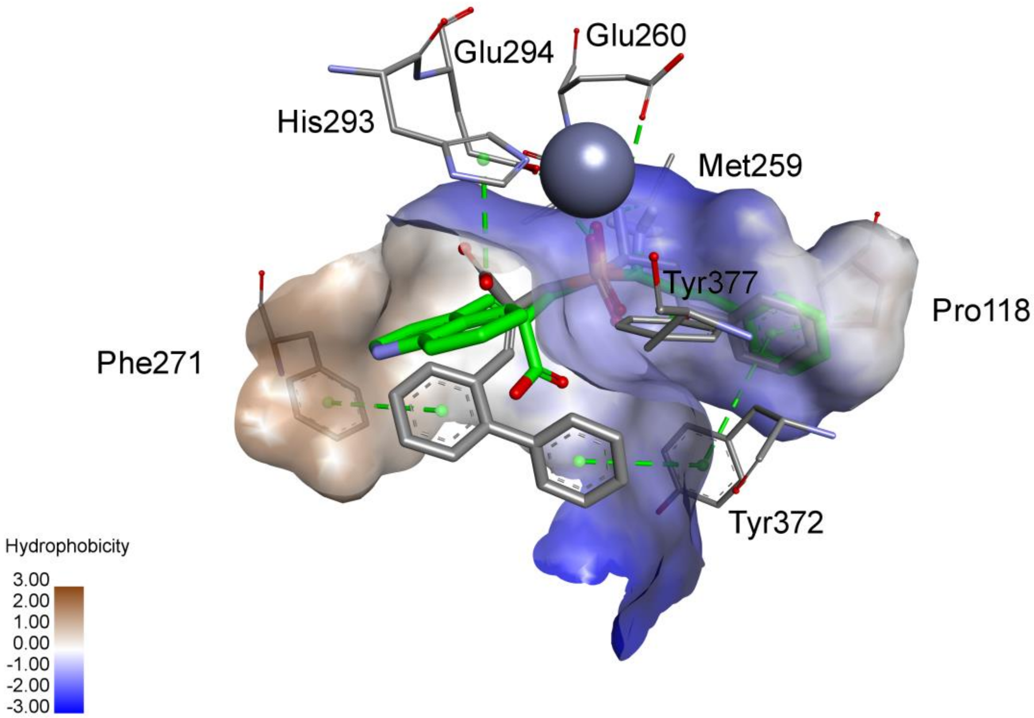

Figure S3. Modeled binding mode of a phosphinic dipeptide analog containing quinolin-4-yl (carbon atoms are colored light green) and biphenyl-2-yl (carbon atoms are colored gray) P1′ fragments in the active center of *Neisseria meningitides* aminopeptidase (PDB: 2GTQ). Inhibitor and enzyme amino acid residues are shown as sticks, while zinc ion as a grey sphere. Hydrogen bonds and non-polar interactions are shown as thin dashed lines. Surface represents the hydrophobicity of side chains the cavity amino acids.

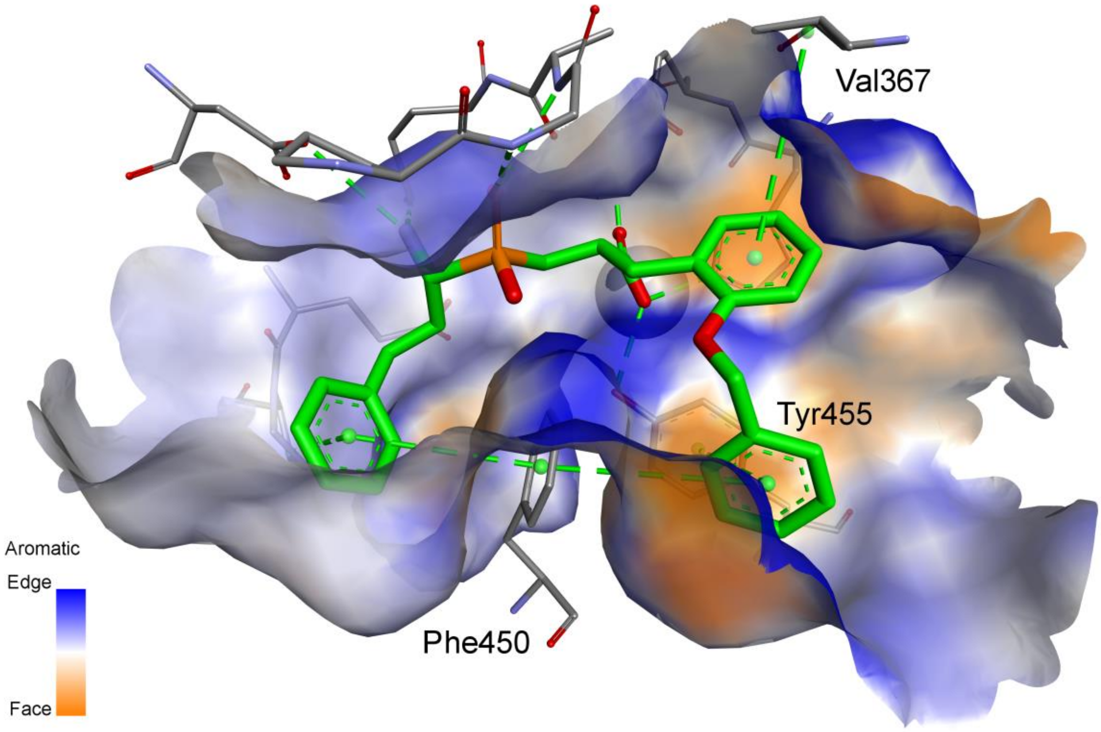

Figure S4. Modeled binding mode of a phosphinic dipeptide analog containing a 2-benzyloxyphenyl P1′ fragment in the active center of ERAP2 aminopeptidase (PDB: 5AB0). Inhibitor and enzyme amino acid residues are shown as sticks, while zinc ion as a grey sphere. Hydrogen bonds and non-polar interactions are shown as thin dashed lines. Surface represents the type of possible aromatics interactions with the side chains of the cavity amino acids.

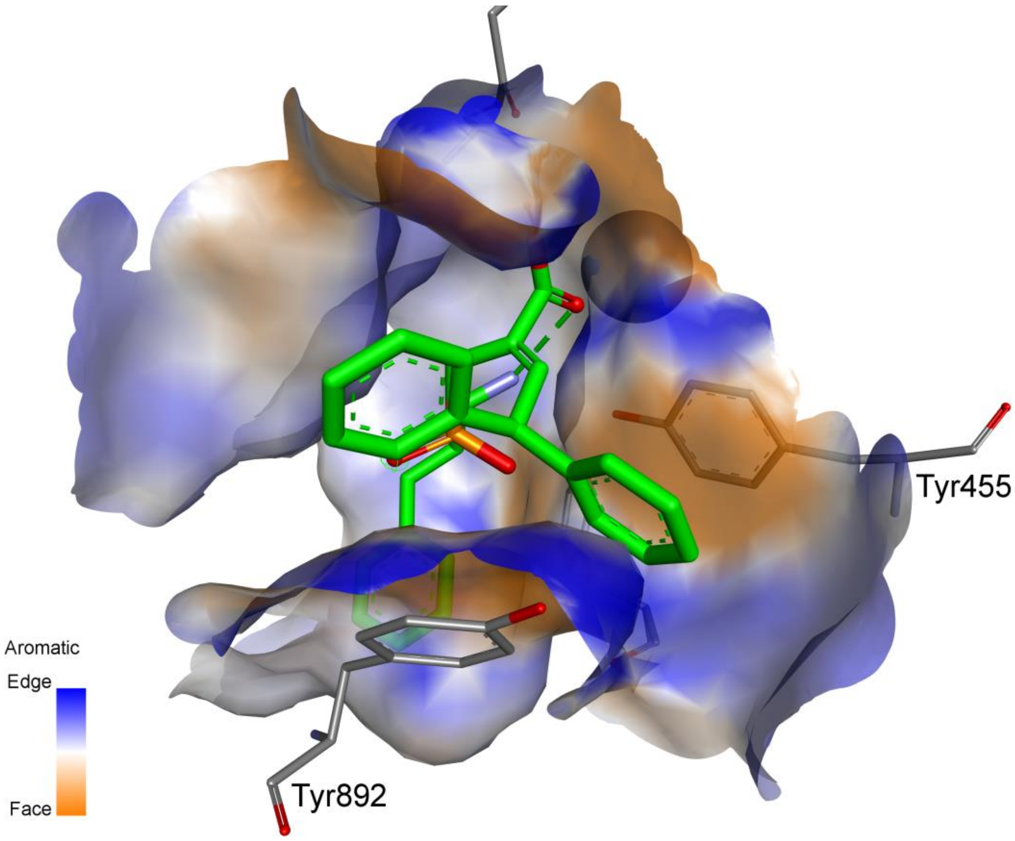

Figure S5. Modeled binding mode of a phosphinic dehydrodipeptide analog containing a diphenylmethyl P1′ fragment in the active center of ERAP2 aminopeptidase (PDB: 5AB0). Inhibitor and enzyme amino acid residues are shown as sticks, while zinc ion as a grey sphere. Hydrogen bonds and non-polar interactions are shown as thin dashed lines. Surface represents the type of possible aromatics interactions with the side chains of the cavity amino acids.

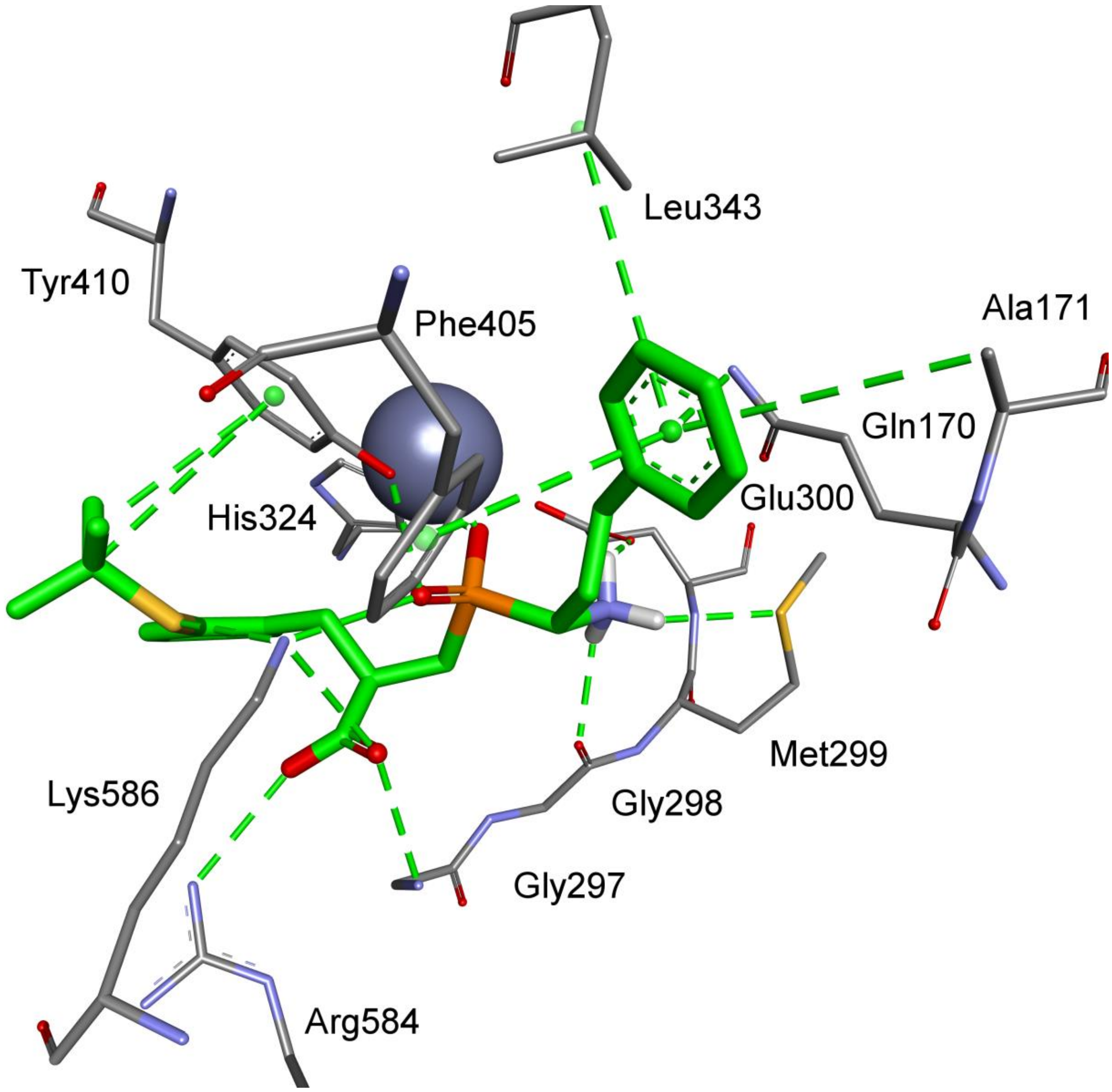

Figure S6. Modeled binding mode of a phosphinic dipeptide analog containing a 2-*tert*-butylthiophenyl P1′ fragment in the active center of cold-active aminopeptidase (PDB: 5AB0). Inhibitor and enzyme amino acid residues are shown as sticks, while zinc ion as a grey sphere. Hydrogen bonds and non-polar interactions are shown as thin dashed lines.

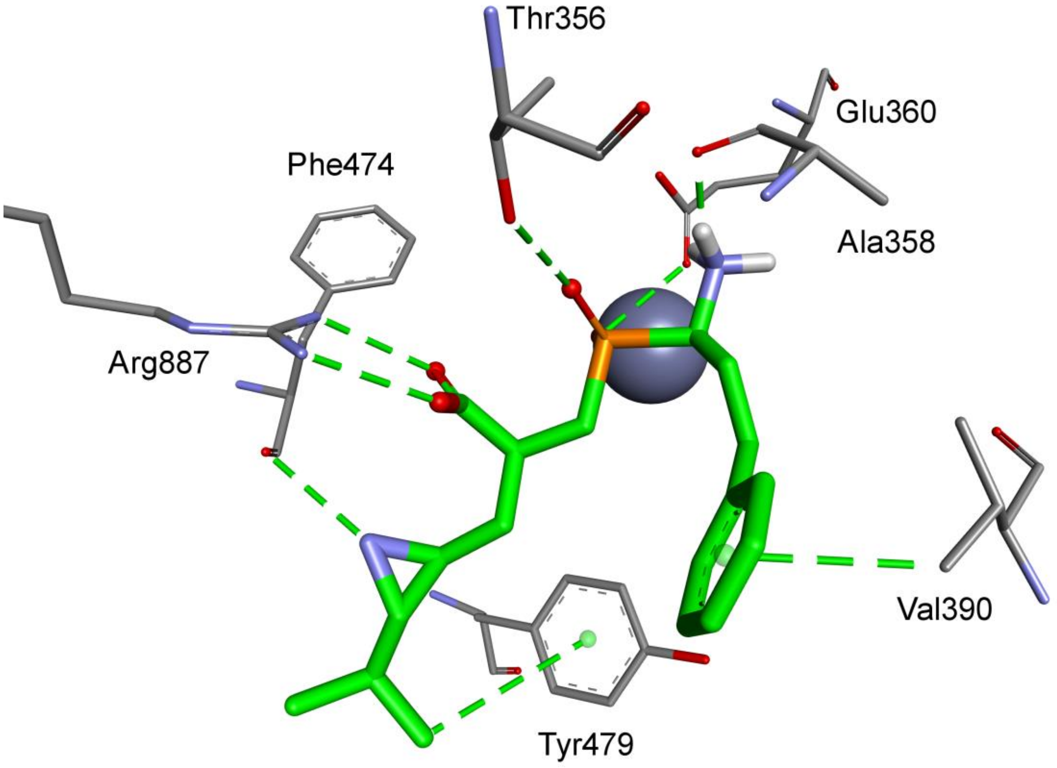

Figure S7. Modeled binding mode of a phosphinic dipeptide analog containing a 3-isobutylaziridin-2-yl P1′ fragment in the active center of Aminopeptidase A (PDB: 4KX7). Inhibitor and enzyme amino acid residues are shown as sticks, while zinc ion as a grey sphere. Hydrogen bonds and non-polar interactions are shown as thin dashed lines.

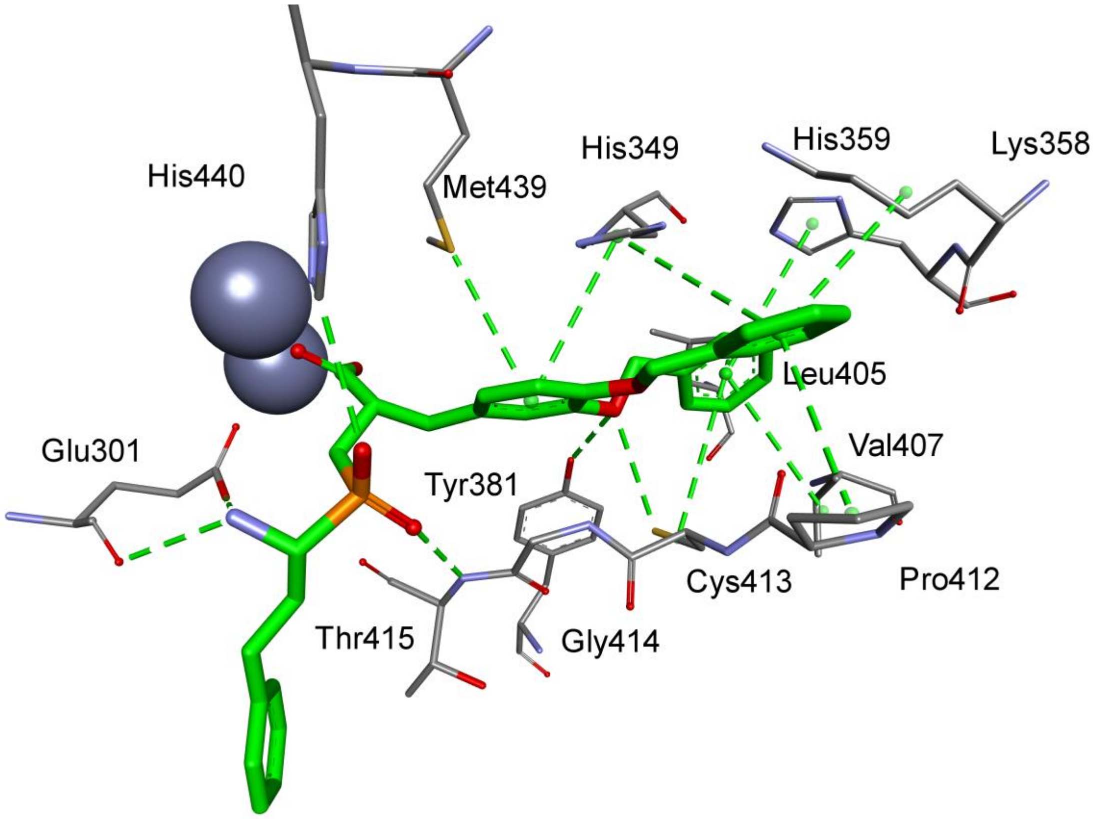

Figure S8. Modeled binding mode of a phosphinic dipeptide analog containing a 3,4-dibenzyloxyphenyl P1′ fragment in the active center of aspartyl aminopeptidase (PDB: 4DYO). Inhibitor and enzyme amino acid residues are shown as sticks, while zinc ions as a grey spheres. Hydrogen bonds and non-polar interactions are shown as thin dashed lines.

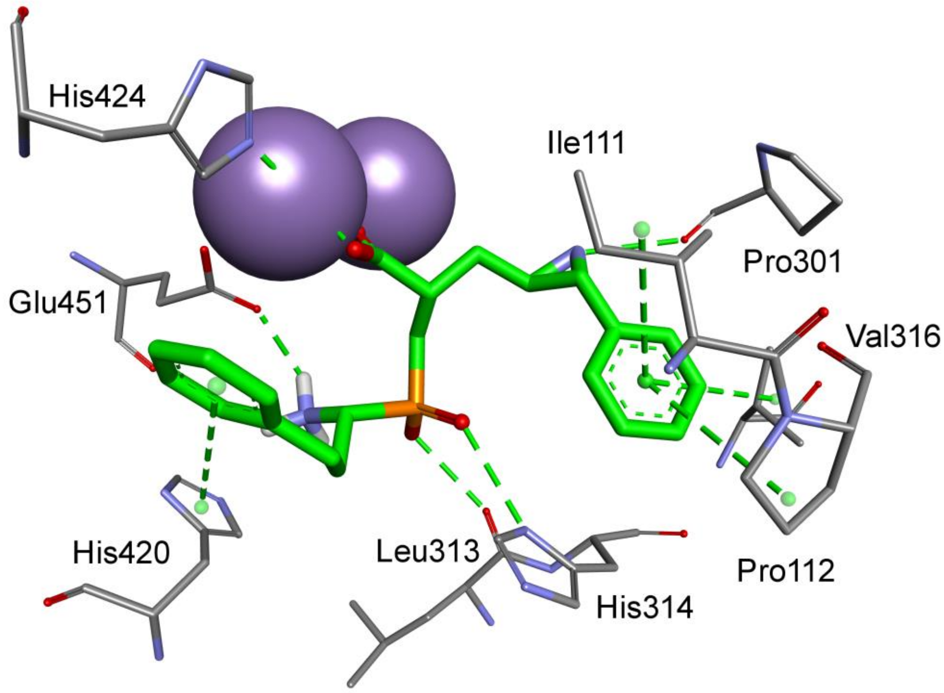

Figure S9. Modeled binding mode of a phosphinic dipeptide analog containing a 3-phenylaziridin-2-yl P1′ fragment in the active center of aminopeptidase P3 (PDB: 5X49). Inhibitor and enzyme amino acid residues are shown as sticks, while manganese ions as a purple spheres. Hydrogen bonds and non-polar interactions are shown as thin dashed lines.

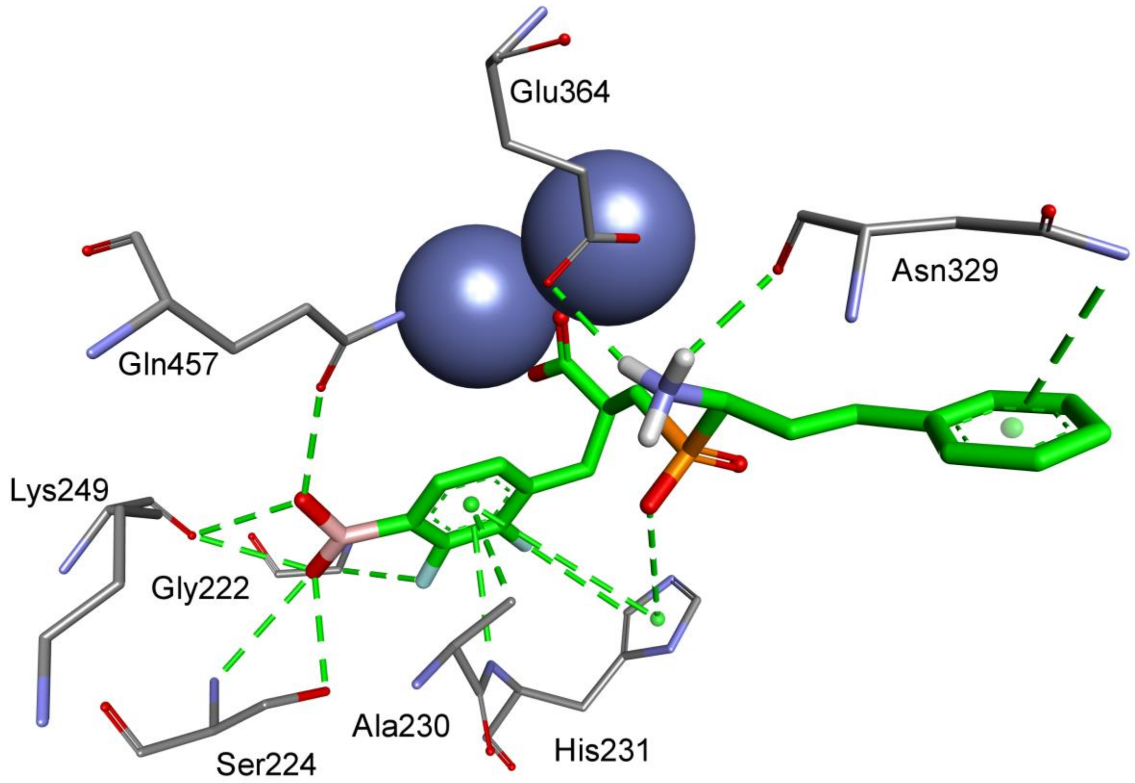

Figure S10. Modeled binding mode of a phosphinic dipeptide analog containing a 4-borono-2,3-difluorophenyl P1′ fragment in the active center of methionyl aminopeptidase 2 (PDB: 1B6A). Inhibitor and enzyme amino acid residues are shown as sticks, while cobalt ions as a purple spheres. Hydrogen bonds and non-polar interactions are shown as thin dashed lines.

S5. References

1. Vassiliou, S.; Węglarz-Tomczak, E.; Berlicki, Ł.; Pawełczak, M.; Nocek, B.; Mulligan, R.; Joachimiak, A.; Mucha, A. Structure-guided, single-point modifications in the phosphinic dipeptide structure yield highly potent and selective inhibitors of neutral aminopeptidases. *J. Med. Chem.* **2014**, *57*, 8140–8151.
2. Su, L.; Jia, Y.; Zhang, L.; Xu, Y.; Fang, H.; Xu, W. Design, synthesis and biological evaluation of novel amino acid ureido derivatives as aminopeptidase N/CD13 inhibitors. *Bioorg. Med. Chem.* **2012**, 20, 3807–3815.
3. Yang, Y.; Liu, C.; Lin, Y.L.; Li, F. Structural insights into central hypertension regulation by human aminopeptidase A. *J.Biol.Chem.* **2013**, *288*, 25638–25645.
4. Orning, L.; Krivi, G.; Fitzpatrick, F.A. Leukotriene A4 hydrolase inhibition by bestatin and intrinsic aminopeptidase activity establish its functional resemblance to metallohydrolase enzymes. *J. Biol. Chem.* **1991**, *266*, 1375–1378.
5. Fournié-Zaluski, M.C.; Poras, H.; Roques, B.P.; Nakajima, Y.; Ito, K.; Yoshimoto, T. Structure of aminopeptidase N from Escherichia coli complexed with the transition-state analogue aminophosphinic inhibitor PL250. *Acta Crystallogr. Sect. D Biol. Crystallogr.* **2009**, *65*, 814–822.
6. Nikolaou, A.; Eynde, I. Van Den; Tourwé, D.; Vauquelin, G.; Tóth, G.; Mallareddy, J.R.; Poglitsch, M.; Van Ginderachter, J.A.; Vanderheyden, P.M.L. [3H]IVDE77, a novel radioligand with high affinity and selectivity for the insulin-regulated aminopeptidase. *Eur. J. Pharmacol.* **2013**, *702*, 93–102.
7. Papakyriakou, A.; Zervoudi, E.; Tsoukalidou, S.; Mauvais, F.-X.; Sfyroera, G.; Mastellos, D.C.; van Endert, P.; Theodorakis, E.A.; Vourloumis, D.; Stratikos, E. 3,4-Diaminobenzoic acid derivatives as inhibitors of the oxytocinase subfamily of m1 aminopeptidases with immune-regulating properties. *J. Med. Chem.* **2015**, *58*, 1524–1543.
8. Deprez-Poulain, R.; Flipo, M.; Piveteau, C.; Leroux, F.; Dassonneville, S.; Florent, I.; Maes, L.; Cos, P.; Deprez, B. Structure–activity relationships and blood distribution of antiplasmodial aminopeptidase-1 inhibitors. *J. Med. Chem.* **2012**, *55*, 10909–10917.
9. Węglarz-Tomczak, E.; Poręba, M.; Byzia, A.; Berlicki, Ł.; Nocek, B.; Mulligan, R.; Joachimiak, A.; Drag, M.; Mucha, A. An integrated approach to the ligand binding specificity of Neisseria meningitidis M1 alanine aminopeptidase by fluorogenic substrate profiling, inhibitory studies and molecular modeling. *Biochimie* **2013**, *95*, 419–428.
10. Chan, W.W.C. L-Leucinthiol - A potent inhibitor of leucine aminopeptidase. *Biochem. Biophys. Res. Commun.* **1983**, *116*, 297–302.
11. Dong, L.; Cheng, N.; Wang, M.W.; Zhang, J.; Shu, C.; Zhu, D.X. The leucyl aminopeptidase from Helicobacter pylori is an allosteric enzyme. *Microbiology* **2005**, *151*, 2017–2023.
12. Kale, A.; Pijning, T.; Sonke, T.; Dijkstra, B.W.; Thunnissen, A.M. Crystal structure of the leucine aminopeptidase from Pseudomonas putida reveals the molecular basis for its enantioselectivity and broad substrate specificity. *J. Mol. Biol.* **2010**, *398*, 703–714.
13. Kelly, J.A.; Neidle, E.L.; Neidle, A. An aminopeptidase from mouse brain cytosol that cleaves n-terminal acidic amino acid residues. *J. Neurochem.* **1983**, *40*, 1727–1734.
14. Teuscher, F.; Lowther, J.; Skinner-Adams, T.S.; Spielmann, T.; Dixon, M.W.A.; Stack, C.M.; Donnelly, S.; Mucha, A.; Kafarski, P.; Vassiliou, S.; i in. The M18 aspartyl aminopeptidase of the human malaria parasite Plasmodium falciparum. *J. Biol. Chem.* **2007**, *282*, 30817–30826.
15. Chai, S.C.; Ye, Q.Z. Metal-mediated inhibition is a viable approach for inhibiting cellular methionine

aminopeptidase. *Bioorg. Med. Chem. Lett.* **2009**, *19*, 6862–6864.

16. Helgren, T.R.; Chen, C.; Wangtrakuldee, P.; Edwards, T.E.; Staker, B.L.; Abendroth, J.; Sankaran, B.; Housley, N.A.; Myler, P.J.; Audia, J.P.; i in. Rickettsia prowazekii methionine aminopeptidase as a promising target for the development of antibacterial agents. *Bioorg. Med. Chem.* **2017**, *25*, 813–824.
17. Farrell, P.J.; Zopf, C.J.; Huang, H.J.; Balakrishna, D.; Holub, C.; Bilakovics, J.; Fanjul, A.; Matuszkiewicz, J.; Plonowski, A.; Rolzin, P.; i in. Using target engagement biomarkers to predict clinical efficacy of MetAP2 inhibitors. *J. Pharmacol. Exp. Ther.* **2019**, *371*, 299–308.
18. Haldar, M.K.; Scott, M.D.; Sule, N.; Srivastava, D.K.; Mallik, S. Synthesis of barbiturate-based methionine aminopeptidase-1 inhibitors. *Bioorg. Med. Chem. Lett.* **2008**, *18*, 2373–2376.
19. Mitra, S.; Sheppard, G.; Wang, J.; Bennett, B.; Holz, R.C. Analyzing the binding of Co(II)-specific inhibitors to the methionyl aminopeptidases from Escherichia coli and Pyrococcus furiosus. *J. Biol. Inorg. Chem.* **2009**, *14*, 573–585.
20. Douangamath, A.; Dale, G.E.; D'Arcy, A.; Almstetter, M.; Eckl, R.; Frutos-Hoener, A.; Henkel, B.; Illgen, K.; Nerdinger, S.; Schulz, H.; i in. Crystal structures of staphylococcusaureus methionine aminopeptidase complexed with keto heterocycle and aminoketone inhibitors reveal the formation of a tetrahedral intermediate. *J. Med. Chem.* **2004**, *47*, 1325–1328.
21. Alvarado, J.J.; Nemkal, A.; Sauder, J.M.; Russell, M.; Akiyoshi, D.E.; Shi, W.; Almo, S.C.; Weiss, L.M. Structure of a microsporidian methionine aminopeptidase type 2 complexed with fumagillin and TNP-470. *Mol. Biochem. Parasitol.* **2009**, *168*, 158–167.
22. Prechel, M.M.; Orawski, A.T.; Maggiora, L.L.; Simmons, W.H. Effect of a new aminopeptidase P inhibitor, apstatin, on bradykinin degradation in the rat lung. *J. Pharmacol. Exp. Ther.* **1995**, *275*, 1136-1142.
23. Sulpizio, A.C.; Pullen, M.A.; Edwards, R.M.; Louttit, J.B.; West, R.; Brooks, D.P. Mechanism of vasopeptidase inhibitor-induced plasma extravasation: Comparison of omapatrilat and the novel neutral endopeptidase angiotensin-converting enzyme inhibitor GW796406. *J. Pharmacol. Exp. Ther.* **2005**, *315*, 1306–1313.
24. Drinkwater, N.; Sivaraman, K.K.; Bamert, R.S.; Rut, W.; Mohamed, K.; Vinh, N.B.; Scammells, P.J.; Drag, M.; McGowan, S. Structure and substrate fingerprint of aminopeptidase P from Plasmodium falciparum. *Biochem. J.* **2016**, *473*, 3189–3204.
25. Ben-Meir, D.; Spungin, A.; Ashkenazi, R.; Bblumberg, S. Specificity of Streptomyces griseus aminopeptidase and modulation of activity by divalent metal ion binding and substitution. *Eur. J. Biochem.* **1993**, *212*, 107–112.
26. Stamper, C.C.; Bienvenue, D.L.; Bennett, B.; Ringe, D.; Petsko, G.A.; Holz, R.C. Spectroscopic and X-ray crystallographic characterization of bestatin bound to the aminopeptidase from Aeromonas (Vibrio) proteolytica. *Biochemistry* **2004**, *43*, 9620–9628.
27. Dutoit, R.; Brandt, N.; Legrain, C.; Bauvois, C. Functional characterization of two M42 aminopeptidases erroneously annotated as cellulases. *PLoS One* **2012**, *7*, e50639.
